# Empirical evidence that glucan-interacting amino acid side chains within the transmembrane channel collectively facilitate cellulose synthase function

**DOI:** 10.1007/s11103-025-01615-4

**Published:** 2025-07-09

**Authors:** Albert L. Kwansa, Arielle M. Chaves, Joshua T. Del Mundo, Ethan T. Pierce, Esther W. Gomez, Enrique D. Gomez, Candace H. Haigler, Yaroslava G. Yingling, Alison W. Roberts

**Affiliations:** 1 Department of Materials Science and Engineering, North Carolina State University, Raleigh, NC 27695 USA; 2 Department of Biological Sciences, University of Rhode Island, Kingston, RI, 02881 USA; 3 Department of Chemical Engineering, The Pennsylvania State University, University Park, PA 16802 USA; 4 Department of Crop and Soil Sciences and Department of Plant and Microbial Biology, North Carolina State University, Raleigh, NC 27695 USA; 5 Department of Biomedical Engineering, The Pennsylvania State University, University Park, PA 16802 USA; 6 Department of Materials Science and Engineering and Materials Research Institute, The Pennsylvania State University, University Park, PA 16802 USA

**Keywords:** Glycosyltransferase, *Physcomitrium patens*, Moss, Protein–carbohydrate interactions, β-d-glucose, Cellulose microfibril

## Abstract

The fundamental mechanism of cellulose synthesis is widely conserved across Kingdoms and depends on cellulose synthases, which are processive, dual-function, family 2 glycosyltransferases (GT-2). These enzymes polymerize glucose on the cytoplasmic side of the plasma membrane and export the glucan chain to the cell surface through an integral transmembrane (TM) channel. Structural studies of active plant cellulose synthases (CESAs) have revealed interactions between the nascent glucan chain and the side chains of polar, charged, and aromatic amino acid residues that line the TM channel. However, the functional consequences of modifying these side chains have not been tested *in vivo* in CESAs or other processive GT-2s. To test this, we used an established *in vivo* assay based on genetic complementation of CESA5 in the moss, *Physcomitrium patens.* For accurate prediction of glucan-interacting amino acid residues, we generated a complete homotrimeric molecular model of PpCESA5 using a combination of homology and *de novo* modeling. All-atom molecular dynamics-based analyses of contact metrics and interaction energy identified 23 amino acid residues with high propensity to interact with the nascent glucan chain within the TM channel or on the apoplastic surface of PpCESA5. Mutating any one of 18 of these amino acid residues to alanine, thereby removing their side chains, abolished or impaired CESA function, with the strongest effects observed upon the loss of charged amino acid side chains. This provides direct evidence to support the hypothesis that multiple amino acid residues collectively maintain a smooth energy landscape within the TM channel to facilitate glucan translocation.

**Supplementary Information:**

The online version contains supplementary material available at 10.1007/s11103-025-01615-4.

## Key message

Cellulose synthase function was impaired when transmembrane glucan-interacting amino acids predicted by simulation-based contact analysis were mutated individually, indicating that they collectively maintain the energy landscape for glucan translocation.

**Supplementary Information:**

The online version contains supplementary material available at 10.1007/s11103-025-01615-4.

## Introduction

Cellulose is produced by organisms from all major branches of the tree of life, but the cellulose produced by land plants dominates Earth’s ecosystems and human enterprise. The enzymes that synthesize the cellulose polymer are integral plasma membrane proteins that polymerize β-d-glucose on the cytoplasmic side and export the resulting glucan chains to the apoplastic side through a transmembrane (TM) channel surrounded by transmembrane helices (TMHs). The cellulose synthases of land plants (CESAs) form 18-member assemblies that produce and export glucan chains in close proximity (Penttila and Paajanen 2024; Nixon et al. 2016; Purushotham et al. 2020; Cosgrove et al. 2024). The structure of this multimeric cellulose synthesis complex (CSC) facilitates the assembly and crystallization of a microfibril that is exceptionally strong and recalcitrant to chemical and enzymatic breakdown (Haigler and Roberts 2019; Carpita and McCann 2020). The stability of these microfibrils explains the mass storage of carbon in cellulosic biomass, the strength of cellulose-based biomaterials, and the intractability of cellulose as a source of biofuels. Thus, the ability to modify cellulose microfibril properties through the biosynthetic process could pave the way for developing biomaterials with more diverse uses and less recalcitrant biofuel feedstocks.

The CSCs of seed plants are obligate hetero-oligomers comprising three distinct CESA isoforms (McFarlane et al. 2014), and microfibril assembly depends on the coordination of glucan chain synthesis and export among these isoforms. Thus, modifying the function of one of the three CESA isoforms could alter microfibril assembly (Rongpipi et al. 2024), potentially resulting in desirable properties. Amino acid residues that interact with the nascent glucan chain within the TM channel or on the apoplastic side of the plasma membrane are potential targets for these modifications (Guidi et al. 2023). In general, proteins interact with carbohydrates through hydrophobic carbon–hydrogen-pi (CH-π) stacking of aromatic amino acid side chains with the axial C–H bonds of the sugar rings and hydrogen bonding of polar amino acid side chains with sugar equatorial hydroxyl groups (Gabius et al. 2011; Quiocho 1988). Many of these interactions are dynamic, as illustrated by the sliding of hydrolases along the surfaces of their substrates, which is thought to be mechanistically similar to the translocation of polysaccharides through TM channels (McNamara et al. 2015; Knott et al. 2014; Gabius et al. 2011).

Cellulose synthases are processive family 2 glycosyltransferases (GT-2), which remain tightly bound to their products through repeated cycles of polymerization (Cantarel et al. 2009). Like other enzymes in this class, including hyaluronan, chitin, alginate, and poly-*N*-acetylglucosamine synthases (reviewed in Bi et al. 2015), cellulose synthases export their product through an integral TM channel that is closely associated with the active site and couples polymer translocation to glucan chain polymerization. The mechanism of cellulose catalysis and translocation is best characterized for the cellulose synthase from *Rhodobacter sphaeroides*, RsBcsA (Morgan et al. 2016, 2014, 2013). Similarities between cellulose synthases from bacteria (BcsA) and plants (CESAs), first suggested on the basis of sequence similarity (Pear et al. 1996), have been confirmed by computational modeling (Sethaphong et al. 2013) and cryo-EM structures (Purushotham et al. 2020; Zhang et al. 2021). All have active sites where the acceptor glucan and the donor uridine diphosphate (UDP)-glucose are coordinated by conserved amino acid residues and interact with a finger helix and gating loop, which play central roles in catalysis and translocation (Purushotham et al. 2020; Zhang et al. 2021; Morgan et al. 2016, 2013; Omadjela et al. 2013). The acceptor site is the only position where the polymer is tightly bound with all hydroxyl groups involved in hydrogen bonding (Morgan et al. 2014), and this has been suggested to facilitate movement of each newly added glucose to this site (Knott et al. 2016; Zimmer 2019) and prevent premature release or backsliding of the polymer (Knott et al. 2016).

The roles of protein–glucan interactions within the TM channel remain poorly understood. With the acceptor tightly bound at the terminal glucose unit and the motive force for translocation transmitted through the finger helix, amino acid residues lining the TM channel may minimize energetic costs for smooth translocation of the polymer within the channel (reviewed by McNamara et al. 2015; Knott et al. 2016; Zimmer 2019). However, these channel-lining residues have also been proposed to help prevent dissociation of the acceptor after catalysis (McNamara et al. 2015). The experimentally determined structures of active bacterial and plant cellulose synthases provide snapshots of the interactions between the nascent glucan chain and amino acid residues within the TM channel (Morgan et al. 2013; Purushotham et al. 2020; Zhang et al. 2021; Verma et al. 2023). In cellulose synthases from bacteria (RsBcsA), poplar (PttCESA8), and cotton (GhCESA7), the channel is lined with aromatic side chains positioned to interact with the faces of the glucopyranose rings through CH-π stacking interactions, while the hydrophilic side chains are positioned to interact with equatorial hydroxyl groups of the glucan chain. Some of these amino acid residues are conserved between bacteria and plants (Purushotham et al. 2020; Knott et al. 2016; Slabaugh et al. 2014). However, the effects of modifying these interactions on the *in vivo* function of CESAs or other dual function, processive glycosyltransferases has not been tested (Guidi et al. 2023).

We have previously used the moss *Physcomitrium patens* to test the ability of engineered CESAs to function in either hetero-oligomeric CSCs consisting of multiple CESA isoforms or homo-oligomeric CSCs consisting of a single CESA isoform (Scavuzzo-Duggan et al. 2015, 2018; Burris et al. 2021; Li et al. 2022). The assays are based on complementation of *P. patens cesa5* and *cesa5/6/7* knockout (KO) lines, which have an easily scored phenotype where the leafy gametophores fail to develop beyond the early bud stage and colonies grow indefinitely as protonemal filaments (Goss et al. 2012; Li et al. 2022). When constitutively expressed, PpCESA5 rescues both lines, either by forming homo-oligomeric CSCs in the *cesa5/6/7*KO background, or by assembling with PpCESA6 and PpCESA7 in the *cesa5*KO background (Goss et al. 2012; Li et al. 2022). Although some of the other five PpCESAs can rescue *cesa5*KO (Scavuzzo-Duggan et al. 2018), only PpCESA5 rescues *cesa5/6/7*KO. PpCESA5 is also sufficient for gametophore formation when all other PpCESAs are disabled (Li et al. 2022). These results are explained by the ability of PpCESA5 to function as a homo-oligomer, whereas the other PpCESAs can only form hetero-oligomeric CSCs with a compatible PpCESA partner (Li et al. 2022, 2019; Scavuzzo-Duggan et al. 2018; Norris et al. 2017). Among many land plant CESAs, only PpCESA5 has so far been shown to function *in vivo* in the absence of other CESA isoforms (Li et al. 2022), although CESAs from poplar (PttCESA8), cotton (GhCESA7) and soybean (GmCESA1, GmCESA3 and GmCESA6) function as homotrimers *in vitro* (Purushotham et al. 2020; Zhang et al. 2021; Ho et al. 2025). Together, the two assays enable parallel testing of engineered PpCESA5 in homo-oligomeric CSCs, where the full effects of mutations can be revealed, and hetero-oligomeric CSCs, where the integration of defective subunits could lead to changes in cellulose microfibril properties.

Here, we used *Physcomitrium patens* for *in vivo* testing of the effects of modifying CESA-glucan interactions. We employed an all-atom molecular dynamics (MD) simulation and conducted contact analyses to predict the amino acid side chains within the TM channel and on the apoplastic tail of PpCESA5 that interact with the nascent glucan chain. Since no empirical PpCESA5 structure was available, we initially generated a complete homotrimeric model using structural information from an active PttCESA8 homotrimer (Purushotham et al. 2020); a complete, monomeric model of GhCESA1 (Kwansa et al. 2024); and a *de novo* model of the first 29 residues of the longer N-terminal domain of PpCESA5. Each amino acid residue strongly implicated by MD-based contact analysis was mutated to alanine, which eliminates side chain atoms beyond the β-carbon without altering overall protein conformation (Cunningham and Wells 1989; Weiss et al. 2000), and tested for complementation of *cesa*KO mutants. In the *cesa5/6/7*KO background line, where PpCESA5 functions within homo-oligomeric CSCs, loss of single predicted glucan-interacting amino acid side chains abolished or impaired function, consistent with a requirement for a smooth energy profile within the TM channel that can be disrupted by loss of a single interaction. However, when mutations that abolished function in *cesa5/6/7*KO were tested in the *cesa5*KO background line, the resulting gametophores had cell expansion defects suggesting that cell wall properties were altered by integration of defective PpCESA5 subunits into otherwise functional CSCs.

## Materials and methods

### Model generation and contact analysis

#### Model construction

**Fig. 1:**
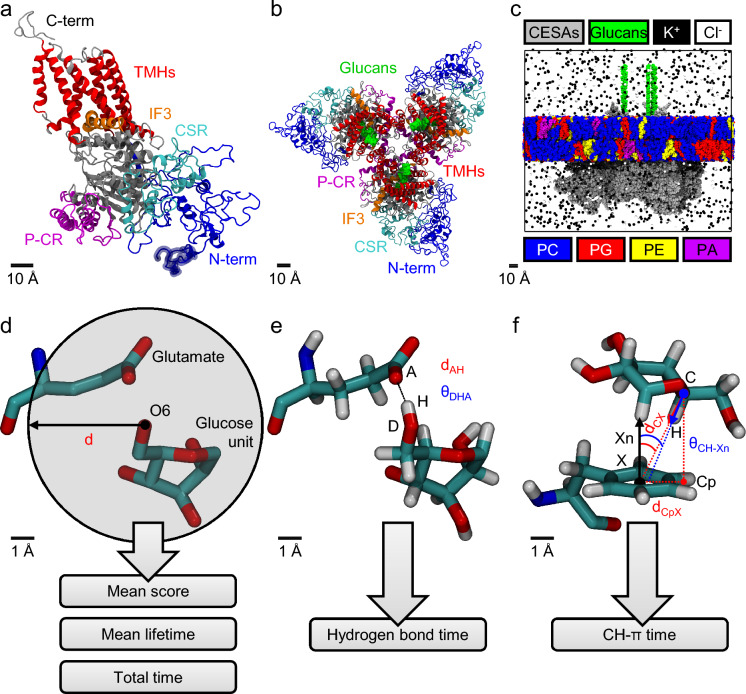
Three-dimensional PpCESA5 protein modeling, system construction, and post-simulation contact analyses. **a** A snapshot of the initial PpCESA5 monomeric model after merging the SWISS-MODEL homology model (30–1081 aa) and the RaptorX-Contact prediction (1–29 aa). Regions of interest are highlighted as indicated. The first 29 residues are indicated with a semi-transparent region at the start of the N-terminal domain. **b** A snapshot of the initial PpCESA5 homotrimeric model after 3D alignment using the cryo-EM PttCESA8 6WLB structure as a reference with 22-mer glucan chains extended from the original cellopentaoses of 6WLB. **c** A snapshot of the initial PpCESA5 homotrimeric model after the addition of a heterogeneous phospholipid bilayer, water, and KCl. The explicit water molecules are hidden for clarity. The system components are highlighted as indicated in the color key. The lower key elements (PC, PG, PE, and PA) represent the lipid polar head groups (phosphatidylcholine, phosphatidylglycerol, phosphatidylethanolamine, and phosphatidic acid, respectively) used to color the entirety of the lipid molecules (head and tail). **d** A schematic of the analysis of protein-glucan contacts based on a distance criterion “d”, which was used to obtain contact metrics including mean score, mean lifetime, and total time. The snapshot shows the heavy atoms only of a β-d-glucose unit of a glucan chain in close proximity to a glutamate residue with a spherical cutoff distance centered at oxygen atom “O6” of the glucose unit. **e** A schematic of the hydrogen bond analysis based on two criteria (a distance and an angle) involving an acceptor heavy atom “A”, a donor heavy atom “D”, and a donor hydrogen atom “H”. These criteria were used to obtain the hydrogen bond time. The snapshot shows all atoms for the same two structures shown in (d). **f** A schematic of the CH-π analysis based on three criteria (two distances and one angle) involving a glucan carbon “C”, a glucan hydrogen “H”, a centroid of an aromatic ring “X”, a vector normal to the aromatic ring “Xn”, and a projection of the glucan carbon onto the aromatic ring plane “Cp”. These criteria were used to obtain the CH-π time. The snapshot shows a β-d-glucose unit of a glucan chain in close proximity to a phenylalanine residue

A full-length 3D model of a PpCESA5 monomer (Fig. 1a) was obtained using a combination of: (a) a homology model via the SWISS-MODEL web server (Waterhouse et al. 2018), based on a full-length model of GhCESA1 (Kwansa et al. 2024), and (b) a 29-aa N-terminal fragment (not shared with GhCESA1) obtained via the RaptorX-Contact web server (Wang et al. 2017). A 3D model of a PpCESA5 homotrimer (Fig. 1b) was then assembled based on a 3D structural alignment of three copies of the PpCESA5 monomer onto the cryo-EM structure of PttCESA8 (PDB ID: 6WLB) (Purushotham et al. 2020) using Visual Molecular Dynamics (VMD) 1.9.3 (Humphrey et al. 1996).

#### Molecular dynamics (MD) simulation

The PpCESA5 homotrimer system was prepared for the all-atom MD simulation with the AMBER 2019 software package (Case et al. 2019) by incorporating additional components: three 22-mer glucan (1,4-linked β-d-glucose) chains, a heterogeneous phospholipid bilayer representing *P. patens* (Resemann et al. 2019; Grimsley et al. 1981), water, and 0.15 M KCl (Fig. 1c). The employed force fields included ff14SB for proteins (Maier et al. 2015), GLYCAM06 for carbohydrates (Kirschner et al. 2008), Lipid17 for lipids (Case et al. 2019), TIP3P for water (Jorgensen et al. 1983), and the “Joung–Cheatham monovalent ion parameters for TIP3P” for ions (Joung and Cheatham 2008). A 10-stage explicit solvent simulation protocol was employed (Lee et al. 2016; Kwansa et al. 2024). The final stage involved 500 ns at 300 K and 1 atm with a 1.0-nm cutoff, a 2-fs timestep, the SHAKE algorithm (Ryckaert et al. 1977), periodic boundary conditions (PBCs), particle-mesh Ewald (PME) (Darden et al. 1993), and Lennard–Jones correction (Case et al. 2019).

#### Structural quality analysis

The ProSA-web server (Wiederstein and Sippl 2007; Sippl 1993), MolProbity web server (Chen et al. 2010; Williams et al. 2018), and ERRAT web server (Colovos and Yeates 1993) were used to analyze several measures of global structural quality, including the ProSA-web z-score, the MolProbity score and related metrics, and the ERRAT quality factor.

#### Contact analysis

Protein–carbohydrate interactions between the PpCESA5 amino acid side chains and the glucan chains were characterized to obtain mean contact scores, mean contact lifetimes, total contact times, and hydrogen bond (H-bond) times, using PyContact 1.0.4 with a distance criterion of 0.5 nm (Scheurer et al. 2018) (Fig. 1d, e). Furthermore, carbon–hydrogen-pi (CH-π) contacts were analyzed using VMD and an in-house script based on criteria described previously (Hudson et al. 2015) (Fig. 1f). Lastly, a linear interaction energy (“lie”) analysis using a 1.2-nm cutoff, with Cpptraj from the AMBER 2021 software package (Case et al. 2021), was performed to obtain contact energy metrics. The total contact time was used to suggest amino acid residue sites within PpCESA5 that could influence protein function, thereby guiding the experimental design for mutagenesis studies. See the Supplementary Materials and Methods for additional details on the computational methods described above.

### Genetic complementation assays in Physcomitrium patens

#### General approach

In these assays, the gametophore-deficient phenotype of *cesa5*KO or *cesa5/6/7*KO is rescued by transformation with a plasmid vector that integrates at an intergenic locus and drives expression of PpCESA5 with a constitutive *Act1* promoter (Goss et al. 2012; Li et al. 2022; Scavuzzo-Duggan et al. 2015).

#### Vector construction

Two different methods were used to introduce mutations into the *PpCESA5* coding sequence. For the PCR fusion method (Scavuzzo-Duggan et al. 2015), primers listed in Table S4 were used to amplify overlapping fragments from a pTHAct1Gate expression clone containing the PpCESA5 coding sequence (Burris et al. 2021). The fragments were fused in a single overlap extension reaction and cloned into pDONR 221 P5-P2 (Invitrogen, Grand Island NY USA) as described previously (Scavuzzo-Duggan et al. 2015). The second method used the Q5 Site-Directed Mutagenesis kit according to the manufacturer’s instructions (New England BioLabs, Ipswich MA USA). Primers designed with the aid of NEBaseChanger (https://nebasechanger.neb.com/) are listed in Table S4. A pDONR 221 P5-P2 entry clone containing the coding sequence of PpCESA5 (Burris et al. 2021) was used as a template for the amplification reactions. To construct complementation vectors, the sequence verified entry clones were transferred to the pTHAct1Gate destination vector, along with an entry clone containing a 3XHA tag in pDONR 221 P1-P5r (Scavuzzo-Duggan et al. 2015).

#### Growing, transforming, and scoring moss lines

*Physcomitrium patens* mutant lines *cesa5/6/7*KO-2 (Li et al. 2022) and *cesa5*KO-3 (Burris et al. 2021) were sub-cultured weekly on basal medium supplemented with ammonium tartrate (BCDAT) (Roberts et al. 2011). For the complementation assays, protoplasts were isolated and transformed with mutated or wild-type PpCESA5 expression vectors or an empty negative control vector. Stable antibiotic-resistant colonies, representing independent transformation events, were arrayed, cultured, and scored for complementation of the no gametophore mutant phenotype (Scavuzzo-Duggan et al. 2015). Transformations varied in the number of independent lines (= colonies) generated. The number of colonies scored per treatment ranged from 39 to 177, pooled from 2 to 3 replicate transformations. Transgene expression was verified by western blot analysis (Scavuzzo-Duggan et al. 2015) when transformation with a mutated vector did not fully rescue the mutant phenotype.

#### Statistical analysis of phenotypes

The 95% confidence intervals of the proportion (colonies with and without gametophores) were calculated using the Wilson Score method (Wilson 1927; Newcombe 1998) as described previously (Scavuzzo-Duggan et al. 2018). A two-tailed Fisher’s Exact Test of Independence (Sokal and Rohlf 1981) was used for statistical hypothesis testing (Scavuzzo-Duggan et al. 2015).

### Morphometric analysis of moss leaves

#### Analysis of leaf cell morphology

The *P. patens* gametophore leaves were photographed with polarized light microscopy using identical optical conditions, and measurements were performed as before (Burris et al. 2021) with the following modifications: the region of interest (ROI) was placed similarly but reduced to 0.07 mm^2^ to allow more leaves (plants) to be measured in approximately the same time. Only cells that were entirely within the ROI were measured. Cells were traced by hand in FIJI (https://imagej.net/Fiji) using the ‘polygon selection’ tool, which yielded shape descriptors. FIJI defines circularity as 4π × [Area]/[Perimeter]^2^ with the range of 1.0 to 0.0 spanning a perfect circle to increasingly elongated shapes.

#### Statistical analysis

Welch’s ANOVA and the Games Howell test were used to analyze differences between groups, including Bonferroni correction for multiple comparisons. The software was Microsoft Excel supplemented with the Real Statistics Resource Pack software (Zaiontz 2019).

### Cell wall analysis by grazing incidence wide-angle X-ray scattering

#### Substrate preparation

Silicon substrates were prepared by cutting bare silicon test grade wafers into ~ 15 × 15 mm squares using a diamond scribe. The wafers were scrubbed with soap and water, then washed sequentially in acetone, isopropanol, and deionized water, dried with an air gun, and then UV-ozone cleaned for 20 min.

#### Mounting moss leaves

Moss gametophores were stored in 2% SDS for 1–2 days to remove intracellular contents and rinsed thoroughly (15 min × 3) in deionized water. Each sample consisted of 10 fully-expanded gametophore leaves collected from 3 to 5 gametophores from an independent culture and laid out flat in a single layer near the center of a silicon substrate. Single leaves were removed from gametophores under a dissecting microscope using two pairs of forceps, one to hold the gametophore at the base and the other to remove the leaf by pulling towards the base. For each genotype, two samples were prepared from each of three lines selected independently from a genetic transformation (n = 3).

#### X-ray scattering measurements

GIWAXS and XRD rocking scan measurements were conducted at beamline 7.3.3, Advanced Light Source, Lawrence Berkeley National Laboratory (Hexemer et al. 2010) using a 10 keV X-ray beam. GIWAXS was measured at a 0.15° incident angle. XRD rocking scans were collected at 5.67°, 6.44°, and 7.23°, corresponding to the (1 $\overline{1}$ 0)/(110) cellulose Iβ reflection. Samples were carefully positioned for XRD rocking scans so that the beam hit all 10 of the leaves. All scans had an exposure time of 30 s. Images were collected using a Pilatus 2 M detector at two different detector heights that were stitched together to remove horizontal gridlines. 2D to 1D data reduction was done using Nika on Igor Pro (Ilavsky 2012).

#### Vertical sector analysis and χ-pole figure reconstruction

GIWAXS vertical cuts, representing the out-of-plane scattering, were obtained by integrating the images at ± 17° from the vertical. The cellulose (200) peak was deconvoluted from the vertical sector cut by fitting the curve as a flat background with the (200) peak, a broad amorphous peak at q ~ 1.4 Å^−1^, and in some samples, a small starch peak at q ~ 1.45 Å^−1^ as a Gaussian. The d-spacing [d(200)] was calculated as d = 2π/q. The coherence length [L(200)] was calculated using the Scherrer equation, L = Kλ/βcos(θ), where K is a shape constant assumed to be 0.9, λ is the X-ray wavelength, β is the full width at half maximum of the peak in radians, and θ is one-half of the scattering angle. χ-pole figures were obtained as previously described (Ye et al. 2020; Baker et al. 2010). Azimuthal intensity profiles of GIWAXS and XRD rocking scans were obtained by integrating over a given q range, 1.0 Å^−1^ < q < 1.3 Å^−1^, corresponding to the (1$\overline{1}$0)/(110) cellulose Iβ reflection. For XRD rocking scans, the azimuthal intensity profiles collected at incident angles of 5.67°, 6.44°, and 7.23° were averaged. The degree of preferred cellulose crystal orientation was assessed using the full width at half maximum of the χ-pole figure. The relative crystalline cellulose content (RCCC) was obtained using the relationship *RCCC* ∝ $\int_{0}^{{\frac{\pi }{2}}} {\sin \left( \upchi \right)} I\left( \upchi \right)d\upchi$ , where χ is the azimuthal angle (Baker et al. 1997; Ye et al. 2020). Biological replicates (n = 3) were lines selected independently from a transformation of *cesa5*KO with a wild-type control or mutated PpCESA5 expression vector. For each biological replicate, spectra from two samples (each consisting of 10 leaves from an independent culture) were collected and analyzed, and the calculated values were averaged prior to statistical analysis. The second sample for one biological replicate of S317A was lost, so values calculated from one measurement were used for statistical analysis.

## Results

### Generation and use of a complete model of a PpCESA5 homotrimer to predict and characterize glucan-interacting amino acid residues

Homology modeling, 3D structure prediction, and 3D alignment were sequentially employed to generate an initial partial-length 1052-aa PpCESA5 monomeric homology model, a full-length 1081-aa PpCESA5 monomeric model (Fig. 1a), and a 3 × 1081-aa PpCESA5 homotrimeric model (Fig. 1b, Online Resource 1). The SWISS-MODEL web server produced two initial homology models based on different target-template sequence alignments. Model 1 (1052 aa: 30 to 1081) was chosen for subsequent use because, compared to Model 2 (1043 aa: 38 to 1080), it covered a greater proportion of the sequence alignment (0.90 vs 0.88) and had a higher Global Model Quality Estimate (GMQE) of 0.56 (vs 0.53), and a more favorable QMEAN z-score of − 5.05 (vs − 5.53). Among 11 3D structure predictions of the fragment needed to complete the N-terminal domain, RaptorX-Contact Model 2 was chosen due to its highest mean rank of 1.7 based on three structural quality metrics (Table S1), including the ERRAT quality factor (ranked 1st, tied), ProSA-web z-score (ranked 1st), and QMEAN z-score (ranked 3rd).

The homotrimeric model was then assembled from three copies of the full-length monomeric model, each consisting of residues 1–29 of the predicted N-terminal domain fragment and the entirety of the homology model. We assessed the structural similarity between this PpCESA5 homotrimer and the PttCESA8 trimeric template (PDB ID: 6WLB, (Purushotham et al. 2020)) used for the trimer 3D alignment stage by calculating three 720-aa α-carbon root-mean-square displacements (RMSDs) between the PpCESA5 homotrimer and the template using corresponding CESA monomers. The mean and standard deviation of these RMSDs are 3.36 ± 0.16 Å (n = 3 monomers).

We evaluated the structural quality of the PpCESA5 homotrimer and analyzed CESA-glucan interactions using the MD simulation trajectory (Fig. 1c, Fig. S1). Global structural quality metrics for the PpCESA5 homotrimer are tabulated along with data from three reference structures: two cryo-EM homotrimeric CESA structures [PttCESA8, PDB ID: 6WLB (Purushotham et al. 2020) and GhCESA7, PDB ID: 7D5K (Zhang et al. 2021)] and one monomeric CESA model [GhCESA1 (Kwansa et al. 2024)]. As mentioned, this PttCESA8 structure served as the trimeric template and this GhCESA1 model served as the monomeric homology modeling template. Notably, the PpCESA5 homotrimer has a ProSA-web z-score of − 8.42 ± 0.45, a MolProbity score of 0.96 ± 0.03 (100th percentile), and an ERRAT quality factor of 89.89 ± 1.76% (Table S2). To provide further context, the average ProSA-web z-score of the PpCESA5 homotrimer is plotted with the ProSA-web z-scores of these three selected reference structures and with the full set of X-ray and NMR structures employed by the ProSA-web server (Fig. S2).

**Fig. 2:**
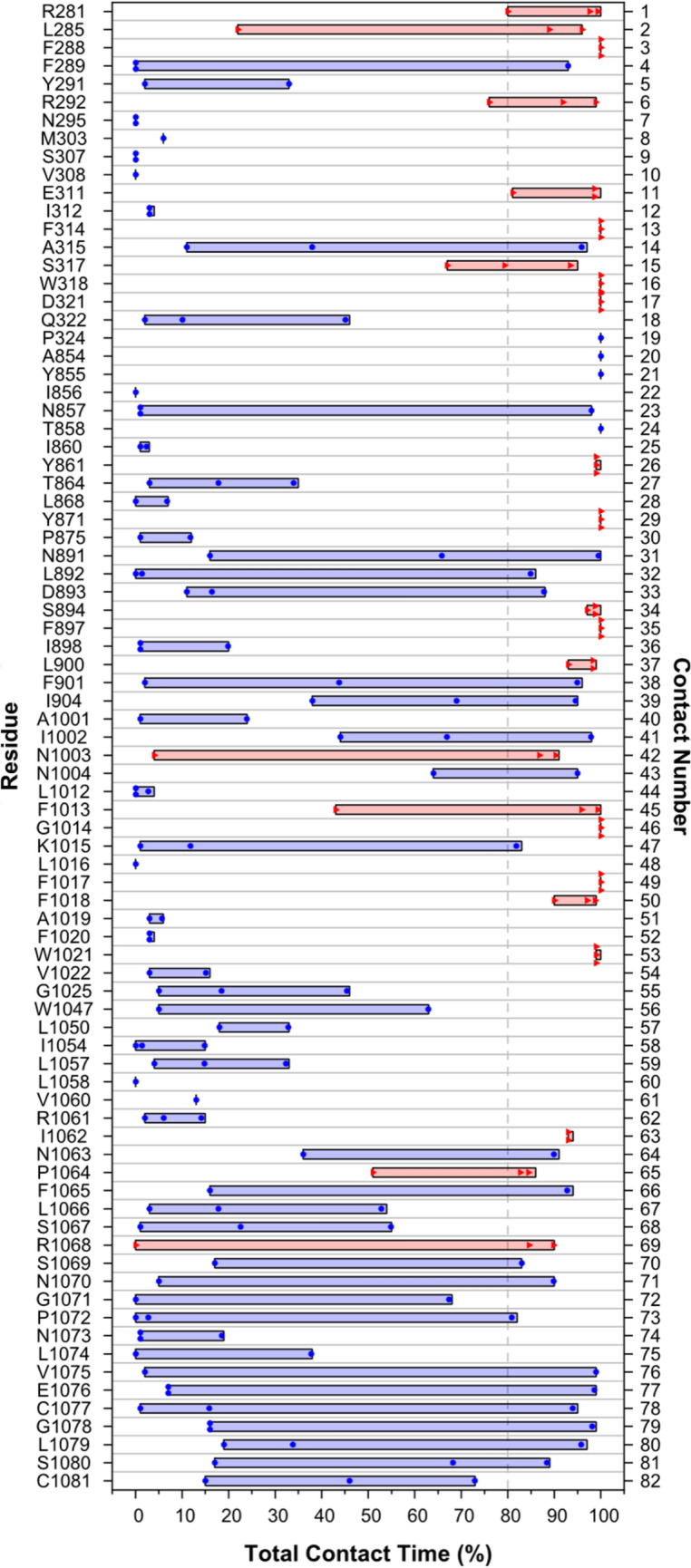
Total contact time for all identified contacts. For each amino acid residue, up to three data points (diamonds) represent contacts involving one of the three glucan chains of the PpCESA5 homotrimer. The red boxes indicate the 23 out of 82 residues for which at least two data points met the criterion for a strong contact (≥ 80%). The blue boxes indicate the 59 out of 82 residues for which these criteria were not met

The analyses of protein–carbohydrate interactions included contact metrics (mean contact score, mean contact lifetime, and total contact time) (Fig. 1d), hydrogen bond time (Fig. 1e), and CH-π time (Fig. 1f). Twenty-three amino acid residues had a total contact time of ≥ 80% for two or more of the three CESAs—the criteria used to identify potential experimental mutation sites (Fig. 2, Table S3). Among those residues located in TMH1-7, which surround the TM channel, and the apoplastic C-terminal domain, these ‘high contact’ residues included three cationic, two anionic, three polar (uncharged), ten aromatic, and five non-polar aliphatic residues (Table S3).

### Functional testing of mutated PpCESA5 in the homo-oligomeric context

To investigate the roles of the predicted glucan-interacting side chains *in vivo*, we mutated each identified amino acid residue to alanine and tested for complementation of *cesa5/6/7*KO, in which the introduced PpCESA5 variant functions within a homo-oligomeric CSC (Li et al. 2022). Each mutated expression vector was transformed in parallel with an unmutated PpCESA5 expression vector (positive control) and an empty vector (negative control). Function was considered abolished (= no rescue) when the proportion of lines with gametophores (vs. all stably transformed lines) did not differ from the negative control; impaired (= partial rescue) when the proportion of lines with gametophores differed from both the negative control and the positive control; or unaffected (= full rescue) when the proportion of lines with gametophores did not differ from the positive control (Fisher’s Exact Test, p < 0.05).

**Fig. 3:**
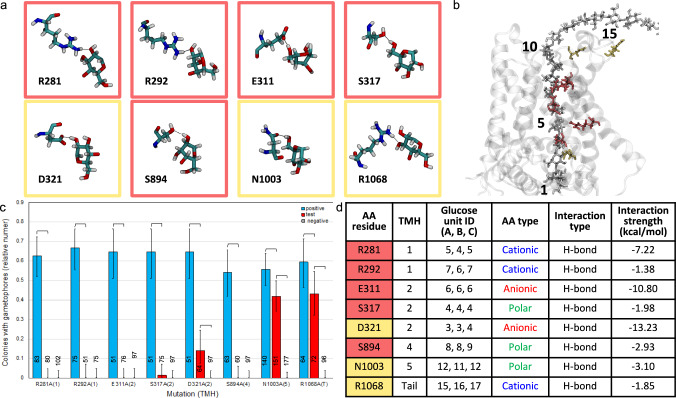
Structure and function of glucan contacts with polar and charged amino acid residues. **a** Contacts between glucose rings and amino acid side chains lining the TM channel of PpCESA5. Boxes are color-coded as red = no rescue or yellow = partial rescue. **b** PpCESA5 TM channel surrounded by TM helices (gray ribbons) and containing a glucan chain (gray stick, numbered starting with the acceptor glucose) with close-contact amino acid residues [stick, color-coded as in (**a**)]. **c** Complementation of *cesa5/6/7*KO by PpCESA5 expression vectors mutated as indicated. Numbers in parentheses indicate the TMH location of the mutations, and “T” indicates the apoplastic tail. Brackets indicate significant differences (p < 0.05, Fisher’s Exact Test) between the test vector and the positive or negative control and numbers at the bottom of each column indicate the number of independent genetic lines scored. **d** Properties of amino acid-glucan contacts. Column A color-coded as in (**a**). Glucose unit IDs are for each of the three CESAs (chains A–C) in the trimer

The MD simulation identified six polar and charged TMH amino acid residues that form hydrogen bonds with the glucan chain (Fig. 3a, b, d). PpCESA5 function was abolished when the R281 (TMH1), R292 (TMH1), E311 (TMH2), S317 (TMH2) or S894 (TMH4) side chain was mutated and impaired when the D321 (TMH2) side chain was mutated (Fig. 3c). Each glucose unit within the TM channel is coordinated by one or more of these side chains except the acceptor and glucose unit #2 (Fig. 3d), which are both coordinated by catalytic domain residues in PttCESA8 (Purushotham et al. 2020) that are conserved in PpCESA5. The N1003A (TMH5) and R1068A (C-terminal) mutations impaired, but did not abolish, PpCESA5 function. The side chains of these two amino acid residues project from the apoplastic surface of the trimer and interact with the glucan chains as they emerge from the TM channel (Fig. 3b). Confirmation of protein expression by western blotting for non-rescuing vectors (Fig. S3) shows that failure to rescue is due to defects in protein function, not lack of expression.

**Fig. 4:**
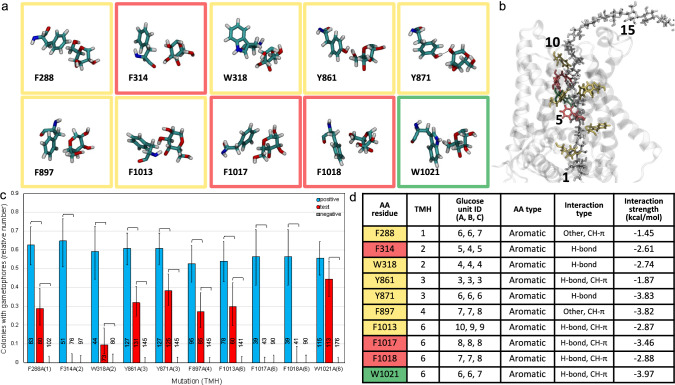
Structure and function of glucan contacts with aromatic amino acid residues. **a** Contacts between glucose rings and amino acid side chains lining the TM channel of PpCESA5. Boxes are color-coded as red = no rescue, yellow = partial rescue, or green = full rescue. **b** PpCESA5 TM channel surrounded by TM helices (gray ribbons) and containing a glucan chain (gray stick, numbered starting with the acceptor glucose) with close-contact amino acid residues [stick, color-coded as in (**a**)]. **c** Complementation of *cesa5/6/7*KO by PpCESA5 expression vectors mutated as indicated. Numbers in parentheses indicate the TMH location of the mutations. Brackets indicate significant differences (p < 0.05, Fisher’s Exact Test) between the test vector and the positive or negative control and numbers at the bottom of each column indicate the number of independent genetic lines scored. **d** Properties of amino acid-glucan contacts. Column A color-coded as in (**a**). Glucose unit IDs are for each of the three CESAs (chains A–C) in the trimer

Ten aromatic TMH amino acid residues were identified by the MD simulation as forming hydrogen bonds and/or CH-π interactions with the glucan chain within the TM channel (Fig. 4a, b, d). Mutation of the F314 (TMH2), F1017 (TMH6) or F1018 (TMH6) side chain abolished PpCESA5 function, whereas mutation of the F288 (TMH1), W318 (TMH2), Y861 (TMH3), Y871 (TMH3), F897 (TMH4) or F1013 (TMH6) side chain impaired function (Fig. 4c). However, PpCESA5 function was not affected by mutation of the W1021 (TMH6) side chain, which coordinates glucose units #6 and #7 along with F288 and Y871 (Fig. 4b, d). Protein expression was confirmed by western blotting for non-rescuing vectors (Fig. S4).

**Fig. 5:**
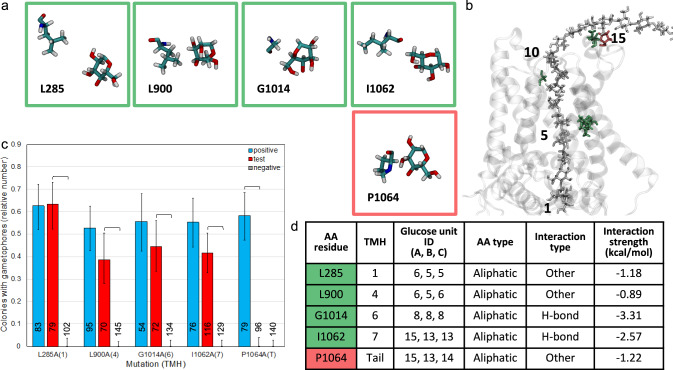
Structure and function of glucan contacts with non-polar aliphatic amino acid residues. **a** Contacts between glucose rings and amino acid side chains lining the TM channel of PpCESA5. Boxes are color-coded as red = no rescue or green = full rescue. **b** PpCESA5 TM channel surrounded by TM helices (gray ribbons) and containing a glucan chain (gray stick, numbered starting with the acceptor glucose) with close-contact amino acid residues [stick, color-coded as in (**a**)]. **c** Complementation of *cesa5/6/7*KO by PpCESA5 expression vectors mutated as indicated. Numbers in parentheses indicate the TMH location of the mutations. Brackets indicate significant differences (p < 0.05, Fisher’s Exact Test) between the test vector and the positive or negative control and numbers at the bottom of each column indicate the number of independent genetic lines scored. **d** Properties of amino acid-glucan contacts. Column A color-coded as in (**a**). Glucose unit IDs are for each of the three CESAs (chains A–C) in the trimer

Of the five non-polar aliphatic amino acid residues identified as glucan-interacting residues by the MD simulation (Fig. 5a, b, d), only P1064 was required for PpCESA5 function (Fig. 5c). P1064, along with I1062, interacts with the glucan chain on the apoplastic surface, outside of the TM channel (Fig. 5b). Protein expression was confirmed by western blotting for P1064A (Fig. S5).

### Functional testing of mutated PpCESA5 in the hetero-oligomeric context

**Fig. 6:**
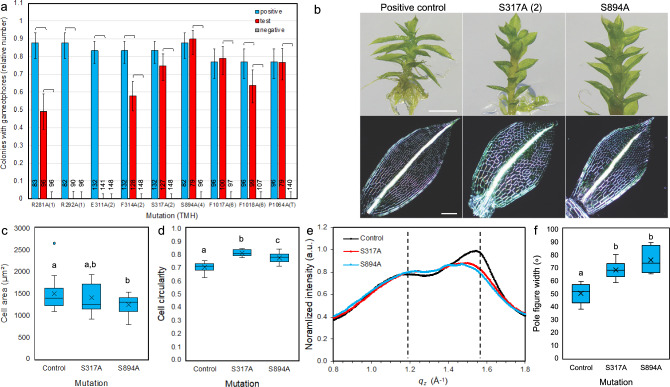
Function of glucan contacts in the *P. patens cesa*5KO background. **a** Complementation of *cesa5*KO by PpCESA5 expression vectors mutated as indicated. Numbers in parentheses indicate the TMH location of the mutations, and “T” indicates the apoplastic tail. Brackets indicate significant differences (p < 0.05, Fisher’s Exact Test) between the test vector and the positive or negative controls and numbers at the bottom of each column indicate the number of independent genetic lines scored. **b** Gametophore morphology (top panels) and leaf structure imaged by polarization microscopy (bottom panels) for *cesa5*KO complemented with wild-type control or mutated PpCESA5 expression vectors. Scale bar for upper panels = 1 mm and scale bar for lower panels = 200 µm. **c** Leaf cell area distributions for control and mutants overlapped, although the mean for S894A was significantly different from the control (p = 0.017). **d** Leaf cell circularity was higher for mutants compared to the control (p < 0.001). **e** GIWAXS intensity versus out-of-plane scattering vector *q*_*z*_ obtained from vertical sector averages (− 17° to 17°) showed qualitative changes to diffraction near the cellulose (200) reflection for S317A and S894A when compared to the control. **f** Full width at half maximum of χ-pole figures [(1 $\overline{1}$ 0)/(110) reflections] showed broadening of χ-pole figures (p = 0.0495) for mutants compared to controls. The pole figure width was similar between S317A and S894A complementation lines (p = 0.62) as contrasted with their differences from controls (p = 0.067 or 0.068). Replication and analysis for C–F were as follows. Biological replicates (n = 3) were lines selected independently from a transformation of *cesa5*KO with a wild-type control or mutated PpCESA5 expression vector. For (**c**) and (**d**), an average of 25.6 cells were measured to obtain mean cell circularity and cell area values for each leaf and values from 7–8 leaves were averaged for each biological replicate. For (**d**), spectra collected from six samples, two each from three biological replicates (a sample was 10 leaves from an independent culture), were averaged for each genotype. For (**f**), values calculated from two samples [as in (**d**)] were averaged for each biological replicate. Welch’s ANOVA followed by the Games Howell test was used to test significant differences between groups: for (**c**), F_(2, 43.7)_ = 4.21; for (**d**), F_(2, 42.0)_ = 75.17; and, for (**f**), F_(2, 3.9)_ = 7.19. Statistically significant differences indicated by different letters on the graphs

To determine how a defective PpCESA5 subunit affects the function of a hetero-oligomeric CSC, we tested all mutations that abolished PpCESA5 function in *cesa5/6/7*KO in *cesa5*KO, in which the engineered PpCESA5 interacts with PpCESA6 and PpCESA7 (Li et al. 2019). Four of these mutations had no effect on the relative number of rescued lines compared to the positive control and two had a significant, but small effect (Fig. 6a). However, mutating S317 (TMH2) or S894 (TMH4) produced a stable complementation phenotype characterized by defects in polarized expansion of the leaf cells and blade (Fig. 6b, c, d). Similar to the effects of a gating loop mutation reported previously (Burris et al. 2021), the gametophores of S317A and S894A complementation lines had wider leaves (Fig. 6b) composed of cells that were similar in area (Fig. 6c), but less elongated (i.e., had higher circularity, Fig. 6d), compared to positive control complementation lines (p < 0.001).

The walls of the leaf cells from the S317A and S894A complementation lines were birefringent as revealed by polarization optics (Fig. 6b), suggesting that cellulose content was not substantially reduced. To test for alteration in nanoscale organization of cellulose, we examined leaves with grazing incidence wide-angle X-ray scattering (GIWAXS) combined with X-ray diffraction (XRD) rocking scans (Rongpipi et al. 2024; Ye et al. 2020; Del Mundo et al. 2023). This approach allows us to decouple the scattering along the plane to scattering orthogonal to the cell wall. Previous work showed that cellulose microfibrils in moss leaves have a preferred crystallographic orientation with the (200) and (1$\overline{1}$0)/(110) planes preferentially stacked parallel to the cell wall plane (Ye et al. 2020). Scattering in the out-of-plane direction for the S317A and S894A leaves showed qualitatively different diffraction profiles near the cellulose (200) reflection (q = 1.55 Å^−1^) compared to the positive control (Fig. 6e, S6a–c) as reported previously for *cesa* mutants in Arabidopsis (Rongpipi et al. 2024). We combined GIWAXS and rocking scan data to create χ-pole figures (Ye et al. 2020), and examined the widths of the pole figures with polar angle as an indication of the orientational organization within the cell wall. The χ-pole figure widths [(1$\overline{1}$0)/(110) reflections] were broadened for the S317A and S894A leaves (Fig. 6f, S6d), consistent with a loss of preferred orientation of cellulose crystals. We found no significant differences between mutants and the positive controls based on the calculated values of relative crystalline cellulose content, d-spacing of the cellulose (200) reflection, and coherence length of the cellulose (200) reflection (Fig. S6e).

Most of the mutations had relatively mild effects on PpCESA5 function when tested in *cesa5*KO (Fig. 6a). However, the R292A (TMH1) and E311A (TMH2) mutations abolished PpCESA5 function, even as part of a hetero-oligomeric CSC with PpCESA6 and PpCESA7 (Fig. 6a). Protein expression was confirmed by western blotting for non-rescuing vectors (Fig. S7).

Whereas the prediction of potential mutation sites focused on the total contact time, additional metrics were used to gain further insights (Fig. S8), including the mean contact score, mean contact lifetime, hydrogen bond time, CH-π time, and contact interaction energies (Coulombic, Lennard–Jones, and total). Among these additional metrics, a high hydrogen bond time and a high total contact interaction energy were the best predictors of the strength of the mutant phenotype (Fig. S9).

## Discussion

### MD simulation combined with genetic complementation assays enables functional testing of predicted mutations

The PpCESA5 homotrimeric model shows good agreement with the cryo-EM PttCESA8 homotrimeric structure (Purushotham et al. 2020). Quantitatively, the α-carbon RMSD between the PpCESA5 model and the PttCESA8 homotrimeric structure (3.36 ± 0.16 Å) is reasonable, given that the initial PpCESA5 homology model was based on a GhCESA1 model (derived partly from the cryo-EM PttCESA8 structure) that had undergone an MD simulation and post-MD energy minimization. It also compares well with the 3.19-Å α-carbon RMSD between this GhCESA1 monomer and the first monomer of this PttCESA8 structure (Kwansa et al. 2024). In the PttCESA8 and GhCESA7 cryo-EM structures, TMH7 from an adjacent CESA contributes to the TM channel (Purushotham et al. 2020; Zhang et al. 2021), and this positioning of TMH7 has also been reported for PpCESA5 from initial cryo-EM results (Massenburg et al. 2024). In contrast, the TM channel is partially exposed to the lipid bilayer in the recent cryo-EM structures of GmCESA1 and GmCESA6 (Ho et al. 2025). This suggests that, for different CESA isoforms, the lipid environment may interact directly and/or indirectly with the product as shown for other transmembrane synthase proteins (Corradi et al. 2018).

Several metrics support the global structural quality of the PpCESA5 trimer model. The ProSA-web z-score of the PpCESA5 model is 0.25 to 0.43 lower than the three reference results, as expected given that the ProSA-web z-score generally decreases as a function of protein length (Fig. S2, Table S2). The value for the PpCESA5 model (− 8.42 ± 0.45) is close to proteins of a similar size, which are underrepresented in the Protein Data Bank compared to smaller proteins (Fig. S2). The MolProbity score of the PpCESA5 model is comparable to the GhCESA1 reference model and lower than those of the two reference cryo-EM structures (Table S2) due to differences in their clashscores (number of atom–atom overlaps per 1000 atoms). The clashscore is zero for the two simulation-based models, which is not unexpected due to the structural geometry optimization and refinement of these models. However, the percentages of poor rotamers, favored rotamers, phi-psi outliers, and phi-psi favored are improved for the cryo-EM structures, with some overlap for phi-psi favored (Table S2). The Ramachandran distribution z-scores are all under the target value (|z|< 2) for the three reference structures and the PpCESA5 model, indicating reasonable backbone dihedral angles (phi and psi) compared to reference proteins from the Protein Data Bank (Hooft et al. 1997). Lastly, the ERRAT quality factor of the PpCESA5 model (89.89 ± 1.76%) is only about 4% lower than the GhCESA1 reference model and within about 6% of a recommended 95% threshold for high-resolution protein structures (Colovos and Yeates 1993).

The present computational and experimental approach suggests that residues in TMHs 1, 2, and 6 have the strongest effects on function among 23 residues that we functionally tested in *P. patens* mutant complementation assays. Amino acid residues with polar and aromatic side chains were over-represented among these 23 residues, as observed for glucan-containing RsBcsA, PttCESA8, and GhCESA7 X-ray and cryo-EM structures (Purushotham et al. 2020; Zhang et al. 2021; Morgan et al. 2016, 2013; Knott et al. 2016) and the binding domains of other classes of proteins that interact with carbohydrates (Hudson et al. 2015). Of the 21 PpCESA5 glucan-interacting residues within the TM channel that were predicted (Table S3, Fig. 2, Fig. S10), 12 align with putative glucan-interacting residues identified in PttCESA8 (Purushotham et al. 2020), and 10 align with residues identified in GhCESA7 (Zhang et al. 2021) (Fig. S10). Eight residues that align across sequences of cellulose synthases in RsBcsA and plants (Fig. S10) also provide evidence of broad evolutionary conservation of the translocation mechanism. These include Y861 (TMH3), which aligns with F416 in RsBcsA. Both form CH-π stacking contacts with glucose unit #3 (Fig. 4a, d), and F416 has been shown to force the glucan chain into a planar conformation as it enters the RsBcsA TM channel (Knott et al. 2016). Other residues conserved across RsBcsA and plants include Y871 (TMH3, aligns with RsBcsA F426) and F1018 (TMH6, aligns with RsBcsA Y558), which interact with glucose units #6 and #7, respectively, in RsBcsA (Knott et al. 2016) and *P. patens* (Fig. 4a, d).

### Loss of single polar, charged, or aromatic channel-lining side chains can have strong deleterious effects on a processive, dual-function glycosyltransferase in vivo

In *cesa5/6/7*KO, the engineered CESA must function within homo-oligomeric CSCs in the absence of class B partners (Li et al. 2022). Complementation of the *cesa5/6/7*KO gametophore-deficient phenotype (Figs. 3 and 4), which is associated with reduced cellulose content in gametophore buds (Goss et al. 2012), is inhibited or abolished by loss of single polar, charged, or aromatic side chains, suggesting that multiple side chains collectively facilitate glucan translocation. This provides empirical support for the ‘greasy slide’ hypothesis, originally proposed based on structural and energetic analysis of the maltoporin TM channel (Meyer and Schulz 1997; Schirmer et al. 1995) and later extended theoretically to translocation in cellulose synthases (Zimmer 2019). In the maltoporin TM channel, which facilitates maltodextrin uptake in bacteria, the energy maxima and minima of the CH-π stacking and the hydrogen bonding interactions with maltotriose are offset by a distance equal to one half of a glucose unit, resulting in a smooth energy profile that minimizes the barriers to translocation (Meyer and Schulz 1997). In this scenario, glucan-interacting side chains collectively facilitate translocation, and the loss of even one required participant can cause strong deleterious effects due to disruption of a smooth energy profile within the PpCESA5 TM channel.

An alternative explanation of the mutagenesis results is that interactions that position the acceptor glucan for efficient glucosyl transfer and prevent premature release of the product are weakened by loss of polar, charged, or aromatic channel-lining side chains. However, potential of mean force calculations for the translocation of glucans through the RsBcsA TM channel showed an energy barrier of only 3.1 kcal/mol, indicating that translocation is not a rate-limiting step for cellulose biosynthesis (Knott et al. 2016). The product is most tightly bound through the terminal glucose within the receptor site (Morgan et al. 2014), consistent with reduced energy barriers within the TM channel (McNamara et al. 2015; Knott et al. 2016; Zimmer 2019). The pulling force required to dislodge the cellulose product from an RsBcsAB complex was recently measured with optical tweezers to be greater than 100 pN. The authors suggested that this strong binding force may result from disruption of the lubricating effects of CH-π stacking and hydrogen bonding interactions due to deformation of either the protein or the product (Hilton et al. 2022). Binding energies associated with CH-π contacts (~ 4–8 kcal/mol) and hydrogen bonds (~ 5–6 kcal/mol) have been reported through experimental techniques and electronic structure calculations (Sheu et al. 2003; Houser et al. 2020). Furthermore, such energies can vary depending on the specific atoms involved and the local environment, for example, being lower in a high-dielectric medium such as water due to electrostatic shielding (Sheu et al. 2003). Our MD simulation showed partial hydration of the TM channel, which would be expected to further reduce the CESA-glucan binding energy and potentially facilitate glucan translocation. Taken together, these observations suggest that cellulose is not tightly bound within the TM channel under native conditions.

### Incorporation of a mutated subunit into a hetero-oligomeric complex alters cellulose preferred orientation with respect to the cell wall plane

*Physcomitrium patens* provides the unique opportunity to test how mutated CESAs function in homo-oligomeric CSCs with all subunits equally impaired compared to hetero-oligomeric CSCs in which mutated CESAs function together with wild-type CESAs, potentially leading to synthesis of fewer glucan chains (if only the wild-type CESAs are active) or the same number of glucan chains, but at different rates (if the mutated CESAs synthesize glucan chains more slowly). When mutations that abolished complementation of *cesa5/6/7*KO were tested in *cesa5*KO, gametophore development was partially restored in most cases. Presumably, this occurred through the assembly of partially functional CSCs in which PpCESA6 and PpCESA7 synthesized microfibrils alone or with some contribution of the mutated CESAs. The leaf cells were isodiametrically expanded in the S317A and S894A complementation lines, similar to when *cesa5*KO was rescued by a PpCESA5 gating loop mutant as reported previously along with evidence that the analogous mutation in Arabidopsis partially inhibited the synthesis of crystalline cellulose (Burris et al. 2021). Isodiametric cell expansion is common in cellulose-deficient mutants, including *radial swelling1*, the first CESA mutant described (Arioli et al. 1998). However, the similar birefringence of leaf cell walls in the S317A, S894A and control complementation lines (Fig. 6b) is consistent with similar crystalline cellulose content, leading to further investigation of cellulose properties. Recently, GIWAXS has revealed for the first time that cellulose microfibrils in the cell walls of plant tissues, including *P. patens* leaves, have a preferred orientation with their (200) and (1$\overline{1}$0)/(110) crystallographic planes preferentially stacked parallel to the cell wall plane (Ye et al. 2020). The reduction in preferred orientation in S317A and S894A complementation lines compared to controls (Fig.  6f, S6d) is similar to the *cesa3*^*je5*^ mutant in Arabidopsis (Rongpipi et al. 2024). Loss of preferred cellulose crystal orientation relative to the cell wall plane was correlated with reduced cell elongation in Arabidopsis hypocotyls (Rongpipi et al. 2024) as observed here for moss leaf cells. Taken together, the reductions in polarized leaf cell expansion and preferred cellulose crystallographic orientation, when CESAs with translocation pore mutations are incorporated into CSCs along with fully functional CESAs, highlight the linkage between glucan chain translocation and normal microfibril assembly.

Although we observed qualitative differences in GIWAXS profiles between the S317A and S894A complementation lines and controls near the (200) cellulose reflection (Fig. 6e), there were no significant differences in calculated values for d-spacing or coherence length of this reflection or for relative crystalline cellulose content. Previously reported differences between Arabidopsis genotypes were small for primary cell walls (Rongpipi et al. 2024), and our sample size was limited. Thus, these data do not rule out the possibility that these parameters are subtly altered by incorporation of PpCESA5 with translocation pore mutations into hetero-oligomeric CSCs. Indeed, it was suggested that expansion of the cellulose (200) plane d-spacing observed in the Arabidopsis *cesa3*^*je5*^ mutant could result from incorporation of defective CESA subunits into hetero-oligomeric CSCs (Rongpipi et al. 2024).

### Some previously reported CESA mutations may impact glucan translocation

Some of the mutations we designed and tested in *P. patens* are homologous to previously characterized *cesa* mutations in Arabidopsis, and the phenotypes observed in this seed plant may also be related to altered glucan translocation. These include *eli1-1* (S301F in AtCESA3, corresponding to S317 in PpCESA5), a cellulose-deficient mutant that also exhibits ectopic lignification (Caño-Delgado et al. 2000, 2003), likely as a response to changes in cell wall integrity (Anderson and Kieber 2020). Four others confer herbicide resistance in Arabidopsis, including L286F in AtCESA6 (Huang et al. 2020); *ixr1-5*, R276H in AtCESA3 (Shim et al. 2018); S892N in AtCESA1 (Hu et al. 2016); and *ixr1-1*, G998D in AtCESA3 or *fxr2-1*, G1013R/E in AtCESA1 (Scheible et al. 2001; Shim et al. 2018). These four distinct mutation sites correspond to PpCESA5 L285, R292, S894, and G1014, respectively. Several of these are cellulose deficient in the absence of herbicides (*ixr1-1*, *ixr1-5*, *fxr2-1*; Shim et al. 2018), consistent with functional conservation between *P. patens* and Arabidopsis. Interestingly, the *aegeus* mutation (A903V in AtCESA1; Harris et al. 2012), which confers quinoxyphen resistance and alters cellulose microfibril crystallinity, was identified as the ortholog of glucan-interacting residue Y455 in RsBcsA using sequence-based alignments (Morgan et al. 2013; Slabaugh et al. 2014; Li et al. 2014). However, in structure-based alignments (Purushotham et al. 2020; Fig. S10), Y455 aligns with L900 in PpCESA5, which is homologous to L898 in AtCESA1. Although implicated by MD as a glucan-interacting residue, mutating L900 did not impair PpCESA5 function in our assay, and the orthologous residues in PttCESA8 (L795) and GhCESA7 (L861) were not implicated in glucan interaction (Purushotham et al. 2020; Zhang et al. 2021). Notably, herbicide resistance mutations are found in all regions of CESA proteins (Larson and McFarlane 2021), so it is unknown whether they affect cellulose polymerization, translocation, or other undefined processes required for cellulose synthesis.

## Supplementary Information

Below is the link to the electronic supplementary material.

Supplementary file1 (PDF 6725 kb)

Supplementary file2 (PDB 4041 kb)

## Data Availability

All data are included in the manuscript or supplemental materials.

## References

[CR1] Anderson CT, Kieber JJ (2020) Dynamic construction, perception, and remodeling of plant cell walls. Annu Rev Plant Biol 71:39–69. 10.1146/annurev-arplant-081519-035846 32084323 10.1146/annurev-arplant-081519-035846

[CR2] Arioli T, Peng L, Betzner AS, Burn J, Wittke W, Herth W, Camilleri C, Höfte H, Plazinski J, Birch R, Cork A, Glover J, Redmond J, Williamson RE (1998) Molecular analysis of cellulose biosynthesis in *Arabidopsis*. Science 279:717–720 9445479 10.1126/science.279.5351.717

[CR3] Baker AA, Helbert W, Sugiyama J, Miles MJ (1997) High-resolution atomic force microscopy of native *Valonia* cellulose I microcrystals. J Struct Biol 119:129–138. 10.1006/jsbi.1997.3866 9245753 10.1006/jsbi.1997.3866

[CR4] Baker JL, Jimison LH, Mannsfeld S, Volkman S, Yin S, Subramanian V, Salleo A, Alivisatos AP, Toney MF (2010) Quantification of thin film crystallographic orientation using X-ray diffraction with an area detector. Langmuir 26:9146–9151. 10.1021/la904840q 20361783 10.1021/la904840q

[CR5] Bi Y, Hubbard C, Purushotham P, Zimmer J (2015) Insights into the structure and function of membrane-integrated processive glycosyltransferases. Curr Opin Struct Biol 34:78–86. 10.1016/j.sbi.2015.07.008 26342143 10.1016/j.sbi.2015.07.008 PMC4684724

[CR6] Burris JN, Makarem M, Slabaugh E, Chaves A, Pierce ET, Lee J, Kiemle SN, Kwansa AL, Singh A, Yingling YG, Roberts AW, Kim SH, Haigler CH (2021) Phenotypic effects of changes in the FTVTxK region of an Arabidopsis secondary wall cellulose synthase compared with results from analogous mutations in other isoforms. Plant Direct 5:e335. 10.1002/pld3.335 34386691 10.1002/pld3.335 PMC8341023

[CR7] Caño-Delgado AI, Metzlaff K, Bevan MW (2000) The *eli1* mutation reveals a link between cell expansion and secondary cell wall formation in *Arabidopsis thaliana*. Development 127:3395–3405. 10.1242/dev.127.15.3395 10887094 10.1242/dev.127.15.3395

[CR8] Caño-Delgado A, Penfield S, Smith C, Catley M, Bevan M (2003) Reduced cellulose synthesis invokes lignification and defense responses in *Arabidopsis thaliana*. Plant J 34:351–362. 10.1046/j.1365-313x.2003.01729.x 12713541 10.1046/j.1365-313x.2003.01729.x

[CR9] Cantarel BL, Coutinho PM, Rancurel C, Bernard T, Lombard V, Henrissat B (2009) The carbohydrate-active enzymes database (CAZy): an expert resource for glycogenomics. Nucleic Acids Res 37:D233-238. 10.1093/nar/gkn663 18838391 10.1093/nar/gkn663 PMC2686590

[CR10] Carpita NC, McCann MC (2020) Redesigning plant cell walls for the biomass-based bioeconomy. J Biol Chem 295:15144–15157. 10.1074/jbc.REV120.014561 32868456 10.1074/jbc.REV120.014561 PMC7606688

[CR11] Case DA, Ben-Shalom IY, Brozell SR, Cerutti DS, Cheatham TE III, Cruzeiro VWD, Darden TA, Duke RE, Ghoreishi D, Giambasu G, Giese T, Gilson MK, Gohlke H, Goetz AW, Greene D, Harris R, Homeyer N, Huang Y, Izadi S, Kovalenko A, Krasny R, Kurtzman T, Lee TS, LeGrand S, Li P, Lin C, Liu J, Luchko T, Luo R, Man V, Mermelstein DJ, Merz KM, Miao Y, Monard G, Nguyen C, Nguyen H, Onufriev A, Pan F, Qi R, Roe DR, Roitberg A, Sagui C, Schott-Verdugo S, Shen J, Simmerling CL, Smith J, Swails J, Walker RC, Wang J, Wei H, Wilson L, Wolf RM, Wu X, Xiao L, Xiong Y, York DM, Kollman PA (2019) AMBER 2019. Univeristy of California, San Francisco

[CR12] Case DA, Aktulga HM, Belfon K, Ben-Shalom IY, Brozell SR, Cerutti DS, Cheatham TE III, Cisneros GA, Cruzeiro VWD, Darden TA, Duke RE, Giambasu G, Gilson MK, Gohlke H, Goetz AW, Harris R, Izadi S, Izmailov SA, Jin C, Kasavajhala K, Kaymak MC, King E, Kovalenko A, Kurtzman T, Lee TS, LeGrand S, Li P, Lin C, Liu J, Luchko T, Luo R, Machado M, Man V, Manathunga M, Merz KM, Miao Y, Mikhailovskii O, Monard G, Nguyen H, O’Hearn KA, Onufriev A, Pan F, Pantano S, Qi R, Rahnamoun A, Roe DR, Roitberg A, Sagui C, Schott-Verdugo S, Shen J, Simmerling CL, Skrynnikov NR, Smith J, Swails J, Walker RC, Wang J, Wei H, Wolf RM, Wu X, Xue Y, York DM, Zhao S, Kollman PA (2021) AMBER 2021. University of California, San Francisco

[CR13] Chen VB, Arendall WB III, Headd JJ, Keedy DA, Immormino RM, Kapral GJ, Murray LW, Richardson JS, Richardson DC (2010) MolProbity: all-atom structure validation for macromolecular crystallography. Acta Crystallogr D Biol Crystallogr 66:12–21. 10.1107/S0907444909042073 20057044 10.1107/S0907444909042073 PMC2803126

[CR14] Colovos C, Yeates TO (1993) Verification of protein structures: patterns of nonbonded atomic interactions. Protein Sci 2:1511–1519. 10.1002/pro.5560020916 8401235 10.1002/pro.5560020916 PMC2142462

[CR15] Corradi V, Mendez-Villuendas E, Ingolfsson HI, Gu RX, Siuda I, Melo MN, Moussatova A, DeGagne LJ, Sejdiu BI, Singh G, Wassenaar TA, Delgado Magnero K, Marrink SJ, Tieleman DP (2018) Lipid-protein interactions are unique fingerprints for membrane proteins. ACS Cent Sci 4:709–717. 10.1021/acscentsci.8b00143 29974066 10.1021/acscentsci.8b00143 PMC6028153

[CR16] Cosgrove D, Dupree P, Gomez ED, Haigler CH, Kubicki JD, Zimmer J (2024) How many glucan chains form plant cellulose microfibrils? A mini review. Biomacromol 25:6357–6366. 10.1021/acs.biomac.4c00995 10.1021/acs.biomac.4c00995 PMC11480985 39207939

[CR17] Cunningham BC, Wells JA (1989) High-resolution epitope mapping of hGH-receptor interactions by alanine-scanning mutagenesis. Science 244:1081–1085. 10.1126/science.2471267 2471267 10.1126/science.2471267

[CR18] Darden T, York D, Pedersen L (1993) Particle mesh Ewald: an N⋅log(N) method for Ewald sums in large systems. J Chem Phys 98:10089–10092. 10.1063/1.464397

[CR19] Del Mundo JT, Rongpipi S, Yang H, Ye D, Kiemle SN, Moffitt SL, Troxel CL, Toney MF, Zhu C, Kubicki JD, Cosgrove DJ, Gomez EW, Gomez ED (2023) Grazing-incidence diffraction reveals cellulose and pectin organization in hydrated plant primary cell wall. Sci Rep 13:5421. 10.1038/s41598-023-32505-8 37012389 10.1038/s41598-023-32505-8 PMC10070456

[CR20] Gabius HJ, Andre S, Jimenez-Barbero J, Romero A, Solis D (2011) From lectin structure to functional glycomics: principles of the sugar code. Trends Biochem Sci 36:298–313. 10.1016/j.tibs.2011.01.005 21458998 10.1016/j.tibs.2011.01.005

[CR21] Goss CA, Brockmann DJ, Bushoven JT, Roberts AW (2012) A *CELLULOSE SYNTHASE* (*CESA*) gene essential for gametophore morphogenesis in the moss *Physcomitrella patens*. Planta 235:1355–1367. 10.1007/s00425-011-1579-5 22215046 10.1007/s00425-011-1579-5

[CR22] Grimsley NH, Grimsley JM, Hartmann E (1981) Fatty acid composition of mutants of the moss *Physcomitrella patens*. Phytochemistry 20:151901524. 10.1016/S0031-9422(00)98523-6

[CR23] Guidi C, Biarnes X, Planas A, De Mey M (2023) Controlled processivity in glycosyltransferases: a way to expand the enzymatic toolbox. Biotechnol Adv 63:108081. 10.1016/j.biotechadv.2022.108081 36529206 10.1016/j.biotechadv.2022.108081

[CR24] Haigler CH, Roberts AW (2019) Structure/function relationships in the rosette cellulose synthesis complex illuminated by an evolutionary perspective. Cellulose 26:227–247. 10.1007/s10570-018-2157-9

[CR25] Harris DM, Corbin K, Wang T, Gutierrez R, Bertolo AL, Petti C, Smilgies DM, Estevez JM, Bonetta D, Urbanowicz BR, Ehrhardt DW, Somerville CR, Rose JK, Hong M, Debolt S (2012) Cellulose microfibril crystallinity is reduced by mutating C-terminal transmembrane region residues CESA1A903V and CESA3T942I of cellulose synthase. Proc Natl Acad Sci U S A 109:4098–4103. 10.1073/pnas.1200352109 22375033 10.1073/pnas.1200352109 PMC3306678

[CR26] Hexemer A, Bras W, Glossinger J, Schaible E, Gann E, Kirian R, MacDowell A, Church M, Rude B, Padmore H (2010) A SAXS/WAXS/GISAXS beamline with multilayer monochromator. J Phys 247:012007. 10.1088/1742-6596/247/1/012007

[CR27] Hilton MA, Manning HW, Gorniak I, Brady SK, Johnson MM, Zimmer J, Lang MJ (2022) Single-molecule investigations of single-chain cellulose biosynthesis. Proc Natl Acad Sci U S A 119:e2122770119. 10.1073/pnas.2122770119 36161928 10.1073/pnas.2122770119 PMC9546554

[CR28] Ho R, Pallinti P, Wilson LFL, Wan Y, Zimmer J (2025) Structure, function and assembly of soybean primary cell wall cellulose synthases. Elife. 10.7554/eLife.96704 40365874 10.7554/eLife.96704 PMC12077881

[CR29] Hooft RW, Sander C, Vriend G (1997) Objectively judging the quality of a protein structure from a Ramachandran plot. Comput Appl Biosci 13:425–430. 10.1093/bioinformatics/13.4.425 9283757 10.1093/bioinformatics/13.4.425

[CR30] Houser J, Kozmon S, Mishra D, Hammerova Z, Wimmerova M, Koca J (2020) The CH-π interaction in protein-carbohydrate binding: bioinformatics and in vitro quantification. Chemistry 26:10769–10780. 10.1002/chem.202000593 32208534 10.1002/chem.202000593

[CR31] Hu Z, Vanderhaeghen R, Cools T, Wang Y, De Clercq I, Leroux O, Nguyen L, Belt K, Millar AH, Audenaert D, Hilson P, Small I, Mouille G, Vernhettes S, Van Breusegem F, Whelan J, Hofte H, De Veylder L (2016) Mitochondrial defects confer tolerance against cellulose deficiency. Plant Cell 28:2276–2290. 10.1105/tpc.16.00540 27543091 10.1105/tpc.16.00540 PMC5059812

[CR32] Huang L, Li X, Zhang W, Ung N, Liu N, Yin X, Li Y, McEwan RE, Dilkes B, Dai M, Hicks GR, Raikhel NV, Staiger CJ, Zhang C (2020) Endosidin20 targets the cellulose synthase catalytic domain to inhibit cellulose biosynthesis. Plant Cell 32:2141–2157. 10.1105/tpc.20.00202 32327535 10.1105/tpc.20.00202 PMC7346566

[CR33] Hudson KL, Bartlett GJ, Diehl RC, Agirre J, Gallagher T, Kiessling LL, Woolfson DN (2015) Carbohydrate-aromatic interactions in proteins. J Am Chem Soc 137:15152–15160. 10.1021/jacs.5b08424 26561965 10.1021/jacs.5b08424 PMC4676033

[CR34] Humphrey W, Dalke A, Schulten K (1996) VMD: visual molecular dynamics. J Mol Graph 14:33–38. 10.1016/0263-7855(96)00018-5 8744570 10.1016/0263-7855(96)00018-5

[CR35] Ilavsky J (2012) Nika: software for two-dimensional data reduction. J Appl Crystallogr 45:324–328. 10.1107/S0021889812004037

[CR36] Jorgensen WL, Chandrasekhar J, Madura JD, Impey RW, Klein ML (1983) Comparison of simple potential functions for simulating liquid water. J Chem Phys 79:926–935. 10.1063/1.445869

[CR37] Joung IS, Cheatham TE III (2008) Determination of alkali and halide monovalent ion parameters for use in explicitly solvated biomolecular simulations. J Phys Chem B 112:9020–9041. 10.1021/jp8001614 18593145 10.1021/jp8001614 PMC2652252

[CR38] Kirschner KN, Yongye AB, Tschampel SM, Gonzalez-Outeirino J, Daniels CR, Foley BL, Woods RJ (2008) GLYCAM06: a generalizable biomolecular force field. Carbohydrates J Comput Chem 29:622–655. 10.1002/jcc.20820 17849372 10.1002/jcc.20820 PMC4423547

[CR39] Knott BC, Crowley MF, Himmel ME, Stahlberg J, Beckham GT (2014) Carbohydrate-protein interactions that drive processive polysaccharide translocation in enzymes revealed from a computational study of cellobiohydrolase processivity. J Am Chem Soc 136:8810–8819. 10.1021/ja504074g 24869982 10.1021/ja504074g

[CR40] Knott BC, Crowley MF, Himmel ME, Zimmer J, Beckham GT (2016) Simulations of cellulose translocation in the bacterial cellulose synthase suggest a regulatory mechanism for the dimeric structure of cellulose. Chem Sci 7:3108–3116. 10.1039/C5SC04558D 27143998 10.1039/c5sc04558d PMC4849487

[CR41] Kwansa AL, Singh A, Williams JT, Haigler CH, Roberts AW, Yingling YG (2024) Structural determination of a full-length plant cellulose synthase informed by experimental and *in silico* methods. Cellulose 31:1429–1447. 10.1007/s10570-023-05691-x

[CR42] Larson RT, McFarlane HE (2021) Small but mighty: an update on small molecule plant cellulose biosynthesis inhibitors. Plant Cell Physiol 62:1828–1838. 10.1093/pcp/pcab108 34245306 10.1093/pcp/pcab108

[CR43] Lee J, Cheng X, Swails JM, Yeom MS, Eastman PK, Lemkul JA, Wei S, Buckner J, Jeong JC, Qi Y, Jo S, Pande VS, Case DA, Brooks CL III, MacKerell AD Jr, Klauda JB, Im W (2016) CHARMM-GUI input generator for NAMD, GROMACS, AMBER, OpenMM, and CHARMM/OpenMM simulations using the CHARMM36 additive force field. J Chem Theory Comput 12:405–413. 10.1021/acs.jctc.5b00935 26631602 10.1021/acs.jctc.5b00935 PMC4712441

[CR44] Li X, Speicher TL, Dees D, Mansoori N, McManus JB, Tien M, Trindade LM, Wallace IS, Roberts AW (2019) Convergent evolution of hetero-oligomeric cellulose synthesis complexes in mosses and seed plants. Plant J 99:862–876. 10.1111/tpj.14366 31021018 10.1111/tpj.14366 PMC6711812

[CR45] Li X, Chaves AM, Dees DCT, Mansoori N, Yuan K, Speicher TL, Norris JH, Wallace I, Trindade LM, Roberts AW (2022) Cellulose synthesis complexes are homo-oligomeric and hetero-oligomeric in *Physcomitrella patens*. Plant Physiol 188:2115–2130. 10.1093/plphys/kiac003 35022793 10.1093/plphys/kiac003 PMC8968406

[CR46] Li S, Bashline L, Lei L, Gu Y (2014) Cellulose synthesis and its regulation. The Arabidopsis book/American Society of Plant Biologists 12:e0169. 10.1199/tab.0169 10.1199/tab.0169 PMC3894906 24465174

[CR47] Maier JA, Martinez C, Kasavajhala K, Wickstrom L, Hauser KE, Simmerling C (2015) ff14SB: improving the accuracy of protein side chain and backbone parameters from ff99SB. J Chem Theory Comput 11:3696–3713. 10.1021/acs.jctc.5b00255 26574453 10.1021/acs.jctc.5b00255 PMC4821407

[CR48] Massenburg L, O’Neill H, Nixon BT, Williams AN (2024) Understanding the structural ballet of cellulose formation—using cryoEM to reveal the role of protein flexibility on higher-order oligomer formation, glucan catalysis, and cellulose microfibril extrusion. Microsc Microanal 30:714–718. 10.1093/mam/ozae044.341

[CR49] McFarlane HE, Doring A, Persson S (2014) The cell biology of cellulose synthesis. Annu Rev Plant Biol 65:69–94. 10.1146/annurev-arplant-050213-040240 24579997 10.1146/annurev-arplant-050213-040240

[CR50] McNamara JT, Morgan JL, Zimmer J (2015) A molecular description of cellulose biosynthesis. Annu Rev Biochem 84:895–921. 10.1146/annurev-biochem-060614-033930 26034894 10.1146/annurev-biochem-060614-033930 PMC4710354

[CR51] Meyer JE, Schulz GE (1997) Energy profile of maltooligosaccharide permeation through maltoporin as derived from the structure and from a statistical analysis of saccharide-protein interactions. Protein Sci 6:1084–1091. 10.1002/pro.5560060515 9144780 10.1002/pro.5560060515 PMC2143698

[CR52] Morgan JLW, Strumillo J, Zimmer J (2013) Crystallographic snapshot of cellulose synthesis and membrane translocation. Nature 493:181–186. 10.1038/Nature11744 23222542 10.1038/nature11744 PMC3542415

[CR53] Morgan JL, McNamara JT, Zimmer J (2014) Mechanism of activation of bacterial cellulose synthase by cyclic di-GMP. Nat Struct Mol Biol 21:489–496. 10.1038/nsmb.2803 24704788 10.1038/nsmb.2803 PMC4013215

[CR54] Morgan JL, McNamara JT, Fischer M, Rich J, Chen HM, Withers SG, Zimmer J (2016) Observing cellulose biosynthesis and membrane translocation in crystallo. Nature 531:329–334. 10.1038/nature16966 26958837 10.1038/nature16966 PMC4843519

[CR55] Newcombe RG (1998) Two-sided confidence intervals for the single proportion: comparison of seven methods. Stat Med 17:857–872. 10.1002/(sici)1097-0258(19980430)17:8%3c857::aid-sim777%3e3.0.co;2-e 9595616 10.1002/(sici)1097-0258(19980430)17:8<857::aid-sim777>3.0.co;2-e

[CR56] Nixon BT, Mansouri K, Singh A, Du J, Davis JK, Lee JG, Slabaugh E, Vandavasi VG, O’Neill H, Roberts EM, Roberts AW, Yingling YG, Haigler CH (2016) Comparative structural and computational analysis supports eighteen cellulose synthases in the plant cellulose synthesis complex. Sci Rep 6:28696. 10.1038/srep28696 27345599 10.1038/srep28696 PMC4921827

[CR57] Norris JH, Li X, Huang S, Van de Meene AML, Tran ML, Killeavy E, Chaves AM, Mallon B, Mercure D, Tan H-T, Burton RA, Doblin MS, Kim SH, Roberts AW (2017) Functional specialization of cellulose synthase isoforms in a moss shows parallels with seed plants. Plant Physiol 175:210–222. 10.1104/pp.17.00885 28768816 10.1104/pp.17.00885 PMC5580779

[CR58] Omadjela O, Narahari A, Strumillo J, Melida H, Mazur O, Bulone V, Zimmer J (2013) BcsA and BcsB form the catalytically active core of bacterial cellulose synthase sufficient for in vitro cellulose synthesis. Proc Natl Acad Sci U S A 110:17856–17861. 10.1073/pnas.1314063110 24127606 10.1073/pnas.1314063110 PMC3816479

[CR59] Pear JR, Kawagoe Y, Schreckengost WE, Delmer DP, Stalker DM (1996) Higher plants contain homologs of the bacterial *celA* genes encoding the catalytic subunit of cellulose synthase. Proc Natl Acad Sci U S A 93:12637–12642. 10.1073/pnas.93.22.12637 8901635 10.1073/pnas.93.22.12637 PMC38045

[CR60] Penttila PA, Paajanen A (2024) Critical comment on the assumptions leading to 24-chain microfibrils in wood. Nat Plants 10:1064–1066. 10.1038/s41477-024-01689-w 38769445 10.1038/s41477-024-01689-w

[CR61] Purushotham P, Ho R, Zimmer J (2020) Architecture of a catalytically active homotrimeric plant cellulose synthase complex. Science 369:1089–1094. 10.1126/science.abb2978 32646917 10.1126/science.abb2978

[CR62] Quiocho FA (1988) Molecular features and basic understanding of protein-carbohydrate interactions: the arabinose-binding protein-sugar complex. Curr Top Microbiol Immunol 139:135–148. 10.1007/978-3-642-46641-0_5 3058393 10.1007/978-3-642-46641-0_5

[CR63] Resemann HC, Lewandowska M, Gömann J, Feussner I (2019) Membrane lipids, waxes and oxylipins in the moss model organism *Physcomitrella patens*. Plant Cell Physiol 60:1166–1175. 10.1093/pcp/pcz006 30698763 10.1093/pcp/pcz006 PMC6553664

[CR64] Roberts AW, Dimos C, Budziszek MJ, Goss CA, Lai V (2011) Knocking out the wall: protocols for gene targeting in *Physcomitrella patens*. Methods Mol Biol 715:273–290. 10.1007/978-1-61779-008-9_19 21222091 10.1007/978-1-61779-008-9_19

[CR65] Rongpipi S, Barnes WJ, Siemianowski O, Ye D, Del Mundo JT, Duncombe S, Xin X, Zhu C, Toney MF, Gu Y, Anderson CT, Gomez ED, Gomez EW (2024) Matrix polysaccharides affect preferred orientation of cellulose crystals in primary cell walls. Cellulose 31:1397–1415. 10.1007/s10570-023-05702-x

[CR66] Ryckaert J-P, Ciccotti G, Berendsen HJC (1977) Numerical integration of the cartesian equations of motion of a system with constraints: molecular dynamics of n-alkanes. J Comp Phys 23:327–341. 10.1016/0021-9991(77)90098-5

[CR67] Scavuzzo-Duggan TR, Chaves AM, Roberts AW (2015) A complementation assay for in vivo protein structure/function analysis in *Physcomitrella patens* (*Funariaceae*). App Plant Sci 3:1500023. 10.3732/apps.1500023 10.3732/apps.1500023 PMC4504723 26191463

[CR68] Scavuzzo-Duggan TR, Chaves AM, Singh A, Sethaphong L, Slabaugh E, Yingling YG, Haigler CH, Roberts AW (2018) Cellulose synthase ‘class specific regions’ are intrinsically disordered and functionally undifferentiated. J Integr Plant Biol 60:481–497. 10.1111/jipb.12637 29380536 10.1111/jipb.12637

[CR69] Scheible W-R, Eshed R, Richmond T, Delmer D, Somerville C (2001) Modifications of cellulose synthase confer resistance to isoxaben and thiazolidinone herbicides in *Arabidopsis Ixr1* mutants. Proc Natl Acad Sci USA 98:10079–10084. 10.1073/pnas.191361598 11517344 10.1073/pnas.191361598 PMC56918

[CR70] Scheurer M, Rodenkirch P, Siggel M, Bernardi RC, Schulten K, Tajkhorshid E, Rudack T (2018) PyContact: rapid, customizable, and visual analysis of noncovalent interactions in MD simulations. Biophys J 114:577–583. 10.1016/j.bpj.2017.12.003 29414703 10.1016/j.bpj.2017.12.003 PMC5985026

[CR71] Schirmer T, Keller TA, Wang YF, Rosenbusch JP (1995) Structural basis for sugar translocation through maltoporin channels at 3.1 A resolution. Science 267:512–514. 10.1126/science.7824948 7824948 10.1126/science.7824948

[CR72] Sethaphong L, Haigler CH, Kubicki JD, Zimmer J, Bonetta D, DeBolt S, Yingling YG (2013) Tertiary model of a plant cellulose synthase. Proc Natl Acad Sci U S A 110:7512–7517. 10.1073/pnas.1301027110 23592721 10.1073/pnas.1301027110 PMC3645513

[CR73] Sheu SY, Yang DY, Selzle HL, Schlag EW (2003) Energetics of hydrogen bonds in peptides. Proc Natl Acad Sci U S A 100:12683–12687. 10.1073/pnas.2133366100 14559970 10.1073/pnas.2133366100 PMC240678

[CR74] Shim I, Law R, Kileeg Z, Stronghill P, Northey JGB, Strap JL, Bonetta D (2018) Alleles causing resistance to isoxaben and flupoxam highlight the significance of transmembrane domains for CESA protein function. Front Plant Sci 9:1152. 10.3389/fpls.2018.01152 30197649 10.3389/fpls.2018.01152 PMC6118223

[CR75] Sippl MJ (1993) Recognition of errors in three-dimensional structures of proteins. Proteins 17:355–362. 10.1002/prot.340170404 8108378 10.1002/prot.340170404

[CR76] Slabaugh E, Davis JK, Haigler CH, Yingling YG, Zimmer J (2014) Cellulose synthases: new insights from crystallography and modeling. Trends Plant Sci 19:99–106. 10.1016/j.tplants.2013.09.009 24139443 10.1016/j.tplants.2013.09.009

[CR77] Sokal RR, Rohlf FJ (1981) Biometry. W. H. Freeman and Company, New York

[CR78] Verma P, Kwansa AL, Ho R, Yingling YG, Zimmer J (2023) Insights into substrate coordination and glycosyl transfer of poplar cellulose synthase-8. Structure 31(1166–1173):e1161–e1166. 10.1016/j.str.2023.07.010 10.1016/j.str.2023.07.010 PMC10592267 37572661

[CR79] Wang S, Sun S, Li Z, Zhang R, Xu J (2017) Accurate de novo prediction of protein contact map by ultra-deep learning model. PLoS Comput Biol 13:e1005324. 10.1371/journal.pcbi.1005324 28056090 10.1371/journal.pcbi.1005324 PMC5249242

[CR80] Waterhouse A, Bertoni M, Bienert S, Studer G, Tauriello G, Gumienny R, Heer FT, de Beer TAP, Rempfer C, Bordoli L, Lepore R, Schwede T (2018) SWISS-MODEL: homology modelling of protein structures and complexes. Nucleic Acids Res 46:W296–W303. 10.1093/nar/gky427 29788355 10.1093/nar/gky427 PMC6030848

[CR81] Weiss GA, Watanabe CK, Zhong A, Goddard A, Sidhu SS (2000) Rapid mapping of protein functional epitopes by combinatorial alanine scanning. Proc Natl Acad Sci U S A 97:8950–8954. 10.1073/pnas.160252097 10908667 10.1073/pnas.160252097 PMC16802

[CR82] Wiederstein M, Sippl MJ (2007) ProSA-web: interactive web service for the recognition of errors in three-dimensional structures of proteins. Nucleic Acids Res 35:W407-410. 10.1093/nar/gkm290 17517781 10.1093/nar/gkm290 PMC1933241

[CR83] Williams CJ, Headd JJ, Moriarty NW, Prisant MG, Videau LL, Deis LN, Verma V, Keedy DA, Hintze BJ, Chen VB, Jain S, Lewis SM, Arendall WB III, Snoeyink J, Adams PD, Lovell SC, Richardson JS, Richardson DC (2018) MolProbity: more and better reference data for improved all-atom structure validation. Protein Sci 27:293–315. 10.1002/pro.3330 29067766 10.1002/pro.3330 PMC5734394

[CR84] Wilson EB (1927) Probable Inference, the Law of Succession, and Statistical Inference. J Am Stat Assoc 22:209–212

[CR85] Ye D, Rongpipi S, Kiemle SN, Barnes WJ, Chaves AM, Zhu C, Norman VA, Liebman-Pelaez A, Hexemer A, Toney MF, Roberts AW, Anderson CT, Cosgrove DJ, Gomez EW, Gomez ED (2020) Preferred crystallographic orientation of cellulose in plant primary cell walls. Nat Commun 11:4720. 10.1038/s41467-020-18449-x 32948753 10.1038/s41467-020-18449-x PMC7501228

[CR86] Zaiontz C (2019) Real statistics resource pack software (Release 6.8).

[CR87] Zhang X, Xue Y, Guan Z, Zhou C, Nie Y, Men S, Wang Q, Shen C, Zhang D, Jin S, Tu L, Yin P, Zhang X (2021) Structural insights into homotrimeric assembly of cellulose synthase CesA7 from *Gossypium hirsutum*. Plant Biotechnol J 19:1579–1587. 10.1111/pbi.13571 33638282 10.1111/pbi.13571 PMC8384604

[CR88] Zimmer J (2019) Structural features underlying recognition and translocation of extracellular polysaccharides. Interface Focus 9:20180060. 10.1098/rsfs.2018.0060 30842868 10.1098/rsfs.2018.0060 PMC6388025

