# Supplementary material for: Empirical evidence that glucan-interacting amino acid side chains within the transmembrane channel collectively facilitate cellulose synthase function
Source: Plant Mol Biol. 2025 Jul 9;115(4):85. doi: 10.1007/s11103-025-01615-4 (PMC12241271; doi:10.1007/s11103-025-01615-4)
Supplement: Supplementary file 1 — Supplementary file1 (PDF 6725 kb) [file 11103_2025_1615_MOESM1_ESM.pdf]

Supplemental Information for:

Empirical evidence that glucan-interacting amino acid side chains within the transmembrane channel cooperate to facilitate cellulose synthase function

Albert L. Kwansa[1], Arielle M. Chaves[2], Joshua T. Del Mundo[3], Ethan T. Pierce[4], Esther W. Gomez[3], Enrique D. Gomez[3], Candace H. Haigler[4], Yaroslava G. Yingling[1], Alison W. Roberts[2]*

Corresponding author:

Alison Roberts, Department of Biological Sciences, University of Rhode Island, Kingston, Rhode Island 02881, United States, aroberts@uri.edu.

Supplementary Materials and Methods:

PpCESA5 monomer model construction: An initial PpCESA5 monomer homology model was generated using the amino acid sequence of PpCESA5 (Phytozome ID: Pp3c15_7120V3.1) and a full-length structural template–974-aa GhCESA1 (Kwansa et al. 2024). The GhCESA1 template was a hybrid between its full length, computationally-predicted, structure (Singh et al. 2020) and a partial cryo-EM structure of an ortholog, PttCESA8, (Purushotham et al. 2020). The PpCESA5 target sequence and the GhCESA1 template were input into the SWISS-MODEL web server (Waterhouse et al. 2018), which is based on OpenStructure's ProMod3 (Biasini et al. 2013; Studer et al. 2021). The homology modeling workflow involved a target-template alignment through the HMM-HMM-based lightning-fast iterative sequence search (HHblits) tool (Steinegger et al. 2019). For aligned residue positions, the backbone coordinates of the template were used directly. For non-terminal unaligned residue positions (i.e., internal gaps), structure prediction, using structural fragments from the Protein Data Bank or a Monte Carlo-based approach, was used. Side chains were then rebuilt based on the target sequence, followed by structural energy minimization.

As noted, only aligned residues and internal gaps were modeled in this homology modeling workflow. Thus, any N- or C-terminal unaligned residues (i.e., terminal gaps) were modeled via structure prediction with a separate tool. Three different structure prediction web servers were employed and assessed, namely, I-TASSER (Zhang 2008), Phyre2 (Kelley et al. 2015), and RaptorX-Contact (Wang et al. 2017). The I-TASSER web server uses the Profile-Profile threading Alignment (PPA) and Threading Assembly Refinement (TASSER) methods involving the use of fragment templates from the Protein Data Bank for aligned regions and an ab initio approach for non-aligned regions (Zhang 2008). The Phyre2 web server, when set to "Intensive" mode,

uses a template-based approach and ab initio modeling via the Poing method (Kelley et al. 2015). The RaptorX-Contact web server uses distance and contact matrices with a machine-learning algorithm trained on a set of 6,767 proteins in the Protein Data Bank (length = 26 to 700 aa, identity ≤ 25%, resolution ≤ 0.25 nm) (Wang et al. 2017).

For the PpCESA5 target, there was only one terminal gap, 29 residues at the N-terminal end of the protein. However, since the Phyre2 web server has a lower length limit of 30 residues for the target, the first 30 residues of the sequence were submitted to all three of the chosen structure prediction web servers. I-TASSER and RaptorX-Contact are designed to generate up to five models, while Phyre2 generates one model. All predictions were then assessed with three structural quality web servers, namely, ERRAT (Colovos and Yeates 1993), ProSA-web (Wiederstein and Sippl 2007; Sippl 1993), and QMEAN (Benkert et al. 2011). Structural quality parameters, including ERRAT quality factors, ProSA-web z-scores, and QMEAN4 z-scores, were obtained from each of these web servers.

The selected 30-aa model of the N-terminal PpCESA5 gap was then incorporated into the homology model of PpCESA5 (30 to 1081). Due to the sequence overlap, the entirety of the homology model was used, and only residues 1-29 of the predicted model were used. Using Discovery Studio Visualizer (DSV) (BIOVIA 2021), the predicted model was manually translated so that the carbonyl carbon atom of its residue 29 would be in close proximity to the amine nitrogen atom of residue 30 of the homology model. Then, residue 30 of the predicted model was removed, a peptide bond was formed between residue 29 (predicted model) and residue 30 (homology model), followed by activating a steric bump monitor to track unusually close atoms. In DSV, "steric bumps" are defined as unusually close atom-atom non-bonded distances with a specified van der Waals (VDW) fraction criterion (default of 0.70) (BIOVIA 2021), i.e., if the non-bonded distance between two atoms reaches 70% of their ideal or equilibrium value (sum of their van der Waals radii). The length of the new peptide bond (C_{29}-N_{30}) was then set to 0.1335 nm – the equilibrium value for "C-N" from the AMBER ff14SB force field (Maier et al. 2015). The backbone dihedral angles associated with this peptide bond were set based on manual interactive adjustment (phi and psi) and by incrementing from 0° upward every 15° until all steric clashes were eliminated (omega). This reflects an implementation of one of several possible combinations of the identified backbone dihedral angles.

PpCESA5 homotrimer model construction: Using VMD and in-house Tcl scripting, the full-length (1081 aa) PpCESA5 monomer was triplicated within VMD and structurally aligned, using 720 aa alpha carbon atoms per monomer, to the three monomers of the cryo-EM 6WLB PttCESA8 structure (Purushotham et al. 2020). Secondly, 12 additional glucose units were added from the reducing end of the initial cellodecaose (10-mer) into the apoplastic space using the sequence command of AMBER's tleap; the original cellopentaose (5-mer) from the 6WLB structure was previously extended to 10 repeat units up to the TMH exit using the sculpt tool of Maestro (Singh et al. 2020; Schrödinger 2012). Three dihedral angles of these 12 added glucose units were set to -98.5° (O5-C1-O4'-C4'), -142.3° (C1-O4'-C4'-C5'), and 170.0° (O5-C5-C6-O6), where the single quote indicates the adjacent repeat unit in the reducing end direction; these

three angle values are based on those reported for the origin chain of structure A of cellulose I-beta (Nishiyama et al. 2002).

PpCESA5 homotrimer system assembly: The system was then assembled using AMBER's packmol-memgen (Schott-Verdugo and Gohlke 2019; Martinez et al. 2009) in the context of heterogeneous lipids: 39% DPPC, 19% DAPC, 16% DPPG, 9% DPPE, 8% DAPG, 5% DAPE, 3% DPPA, and 1% DAPA, consisting of fatty acid tail groups (DP = dipalmitoyl and DA = diarachidonyl) and polar head groups (PC = phosphatidylcholine, PG = phosphatidylglycerol, PE = phosphatidylethanolamine, and PA = phosphatidic acid). This lipid composition was based on the mole-percent profiles of polar head groups and fatty acid tail groups that have been experimentally reported for *P. patens* (Resemann et al. 2019; Grimsley et al. 1981) and the set of lipid groups supported by AMBER's Lipid17 force field (Case et al. 2019). Explicit water molecules were added using a buffer distance of 1.5 nm, and K^+ and Cl^- ions were added at 0.15 M. The simulation box was orthogonal with initial dimensions of 23.020 nm, 23.020 nm, and 20.195 nm.

<u>PpCESA5 homotrimer system molecular dynamics simulation</u>: To structurally relax the system and to investigate the dynamics of this protein complex over time, an all-atom molecular dynamics (MD) simulation was then conducted using a 10-stage protocol described previously [1, 2], based in part on CHARMM-GUI (Lee et al. 2016). The system components were represented by the following force fields: ff14SB [16], Lipid17 (Case et al. 2019), TIP3P water model (Jorgensen et al. 1983), and Joung-Cheatham monovalent ion parameters for TIP3P (Joung and Cheatham 2008). The simulation included an energy minimization stage (up to 10,000 steps with 5,000 steepest descent steps followed by conjugate gradient steps), a gradual heating stage (300 K over 100 ps), two stages of NVT equilibration (300 K for 200 ps), five stages of NPT equilibration (300 K and 1 atm for 800 ps), and a final NPT production stage (300 K and 1 atm for 500 ns). NVT and NPT represent thermodynamic ensembles, where N = constant number of particles, V = fixed volume, T = regulated temperature, and P = regulated pressure. For all stages, a 1.0-nm cut-off, periodic boundary conditions (PBCs), particle-mesh Ewald (PME) (Darden et al. 1993), and long-range Lennard-Jones correction were employed (Case et al. 2019). From the heating stage to the production stage, the SHAKE algorithm (Ryckaert et al. 1977) was used to constrain bonds involving hydrogen atoms; the timestep was initially 1.0 fs and was increased to 2.0 fs during the last five stages (four NPT equilibration stages and the production stage). To reduce structural disruptions, harmonic positional restraints were applied and reduced step-wise for the protein atoms (10, 5.0, 2.5, 1.0, 0.5, 0.1 kcal/mol/$Å^2$) and the lipid head group phosphorus atoms (2.5, 2.5, 1.0, 0.5, 0.1, 0.0 kcal/mol/$Å^2$) (Lee et al. 2016). The Langevin thermostat (collision frequency = 1.0 ps^{-1}) and the Berendsen barostat (coupling constant = 1.0 ps, compressibility = $44.6*10^{-6}$ bar^{-1}, anisotropic scaling) were used, where applicable. The CPU-only PMEMD and the GPU-accelerated SPFP PMEMD were used for the energy minimization and subsequent dynamics stages, respectively (Le Grand et al. 2013; Salomon-Ferrer et al. 2013). Exxact Corporation servers equipped with NVIDIA GeForce GTX GPUs were used. Abbreviations: CPU = central processing unit, GPU = graphics processing unit, PMEMD = Particle-Mesh Ewald Molecular Dynamics (simulation program), and SPFP = Single Precision-Fixed Point (mixed precision model).

Structural quality of PpCESA5 model: Several global structural quality metrics were used to assess the PpCESA5 homotrimeric model, following the 500-ns MD simulation and up to 100,000 steps of post-MD energy minimization; each monomer was assessed separately, and then the metrics were averaged over the three monomers.

The ProSA-web server was used to obtain a ProSA-web z-score, which is based on mean force potentials involving alpha carbon atoms and protein-solvent interactions; this metric represents the nativeness of protein folds relative to a set of soluble globular reference structures from the Protein Data Bank (Sippl 1993; Wiederstein and Sippl 2007).

The MolProbity web server was used to obtain several metrics, including: a) a clashscore (number of atom-atom van der Waals overlaps ≥ 0.4 Å per 1,000 atoms), b) percentages of poor rotamers, favored rotamers, phi-psi outliers, phi-psi favored pairs, C-beta deviations, bad bonds, and bad angles, c) a Ramachandran distribution z-score (nativeness of backbone phi-psi dihedral angles relative to a set of reference proteins from the Protein Data Bank), and d) a MolProbity score (approximate resolution calculated as a weighted sum of the clashscore, % of poor rotamers, and % of phi-psi outliers) (Chen et al. 2010; Williams et al. 2018; Hooft et al. 1997; Sobolev et al. 2020). Prior to analysis, the hydrogen atoms were removed and re-added using the "Electron-cloud x-H" method, and Asn/Gln/His sidechain flips were accepted, if proposed.

Lastly, the ERRAT web server was used to obtain an ERRAT quality factor. This metric is calculated as the percentage of 9-residue-long windows with an ERRAT score below a 95% confidence limit, where the ERRAT score is based on the fractions of non-covalent, inter-residue atom-atom contacts (carbon, nitrogen, oxygen/sulfur atoms within 3.5 Å) relative to a set of reference structures from the Protein Data Bank (Colovos and Yeates 1993).

Contact analysis of PpCESA5 molecular dynamics simulation: The prediction of mutation sites was based upon the analysis of contacts between the amino acid residues of the PpCESAs and the beta-D-glucose units of the glucan chains. These predictions were conducted via VMD using an in-house Tcl script to analyze potential contacts involving all atoms of the two selections with a distance ≤ 0.35 nm. This analysis was performed for each of the three CESAs of the homotrimer and their associated glucan chains. If there was at least one atom-atom contact between the CESA residue and the glucose unit, a contact was counted at that time point for the pair of repeat units. A total contact time was then calculated as the number of contacts over time divided by the total number of time points; this analysis was conducted using 100 evenly sampled frames from the 500-ns MD simulation. Then, if a particular CESA-glucan repeat unit pair exhibited a total contact time of ≥ 80% for at least two of the three CESAs, that contact was predicted as a mutation site of interest.

Subsequently, contacts between the amino acid residues of the PpCESAs and beta-D-glucose units of the glucan chains were characterized using PyContact 1.0.4 (Scheurer et al. 2018) using four CPU cores in parallel. First, contacts between heavy atoms of these two selections were identified as those with a distance ≤ 0.5 nm (Fig. 1d); this distance criterion is the default for

PyContact and is supported by previous investigations of protein structure networks (Sobieraj and Setny 2021; Viloria et al. 2017). For each atom-atom contact, a contact score was calculated based on a distance-weighted sigmoidal function [35]. Then, the atom-atom contact scores were accumulated or summed over the residues to obtain residue-residue contact scores, where, in this context, "residue" refers to an amino acid residue or a glucose unit. This contact analysis was conducted over 500 ns of simulated time using 1,000 evenly sampled frames or time points. Then, mean contact scores were calculated by averaging over time. The contact scores over time were also converted into binary values of zero (score = 0) or one (score > 0). This binary contact data was used to obtain contact lifetimes and then mean contact lifetimes. Then, these lifetimes were summed to obtain total contact times. These contact metrics are shown schematically in Fig. 1d.

Hydrogen bond (H-bond) contacts were analyzed based on two criteria, namely, a donor hydrogen-acceptor heavy atom distance (d_{HA}) ≤ 0.25 nm and a donor heavy atom-donor hydrogen-acceptor heavy atom angle (θ_{DHA}) ≥ 120°. This H-bond analysis is shown schematically in Fig. 1e. These criteria were checked for each residue-residue pair, and if there was at least one H-bond for a pair at a given time point, an H-bond contact was counted. Then, after analyzing all residue-residue pairs over time, an H-bond time was calculated for each pair as the number of H-bond contacts over time divided by the total number of time points.

Carbon-hydrogen-pi (CH-π) contacts were analyzed based on three criteria, namely, the distance between a glucan pyranose ring carbon (C) and the geometric center of an aromatic side chain (X) (d_{CX}) ≤ 0.45 nm, the angle between a glucan C-H bond vector and the aromatic ring normal vector (Xn) ($\theta_{CH\text{-}Xn}$) ≤ 40°, and the distance between the position of a glucan pyranose ring carbon projected onto the aromatic ring plane (Cp) and the geometric center of an aromatic side chain (X) (d_{CpX}) ≤ 0.16 nm for histidine (His) or ≤ 0.20 nm for phenylalanine (Phe), tyrosine (Tyr), and the pyranose (6-membered) ring of tryptophan (TrpB). For each residue, aromatic ring plane was defined by two vectors both originating from the ring geometric center and intersecting one of the ring carbon atoms (CG and CD1 for Phe and Tyr, CD2 and CE2 for TrpB). These three criteria are based on those described previously (Hudson et al. 2015). The third criterion (d_{CpX}) was calculated here as $d_{CpX} = d_{CX}*\sin(\theta_{CX\text{-}Xn})$, where $\theta_{CX\text{-}Xn}$ is the angle between a vector from C to X and the aromatic ring plane normal vector. One additional criterion was imposed that the glucan pyranose ring hydrogen atom must be closer to the aromatic side chain geometric center than the glucan pyranose ring carbon atom ($d_{HX} < d_{CX}$). This CH-π contact analysis was conducted with VMD using an in-house Tcl script and is shown schematically in Fig. 1f. These criteria were checked for each pyranose ring C-H bond, and if there was at least one C-H bond within a glucose unit that met these criteria, a CH-π contact was counted for that residue-residue pair at that time point. Then, after analyzing all residue-residue pairs over time, a CH-π time was calculated for each pair as the number of CH-π contacts over time divided by the total number of time points.

Interaction energies associated with the identified contacts were calculated using the linear interaction energy, "lie", action of AMBER 2021's Cpptraj with a 1.2-nm cutoff (default) while accounting for periodic boundary conditions (Åqvist et al. 2002; Case et al. 2021). Three energy

metrics were calculated, namely, the Coulombic energy (E_{Coul}; due to permanent-dipole interactions), the 12-6 Lennard-Jones energy (E_{LJ}; due to induced-dipole interactions or van der Waals attraction and Pauli exclusion), and the total non-bonded energy (E_{Total}; the sum of E_{Coul} and E_{LJ}).

Supplementary Figures:

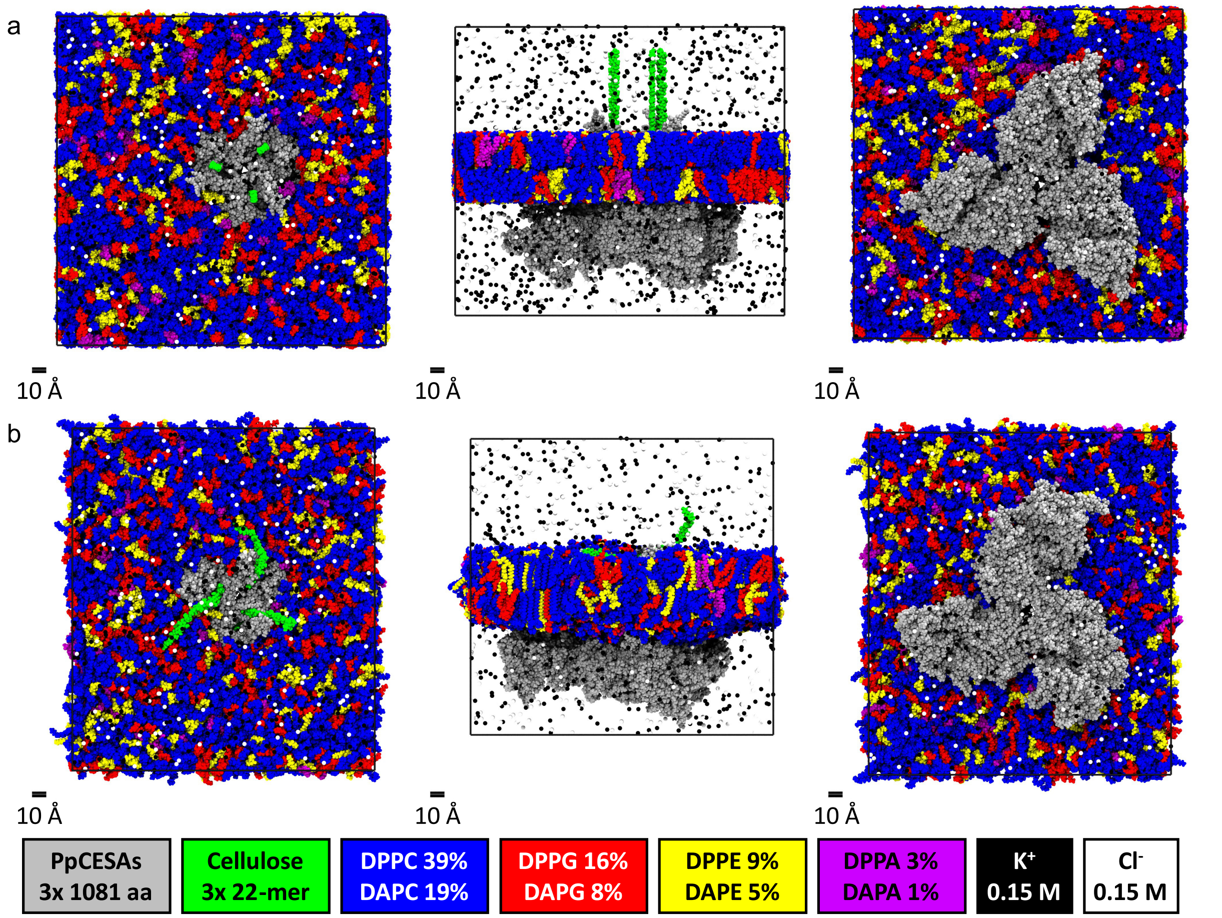

Fig. S1: PpCESA5 homotrimer system. a) Snapshots of the initial PpCESA5 homotrimer model after the addition of a heterogeneous phospholipid bilayer, water, and 0.15 M KCl. Top (apoplastic), side, and bottom (cytosolic) views are shown at the left, middle, and right, respectively. The explicit water molecules are hidden here for clarity. The system components are highlighted as indicated in the color key. b) Snapshots of the refined PpCESA5 homotrimer model after conducting a 500-ns all-atom MD simulation. The system is visually represented as described in (a).

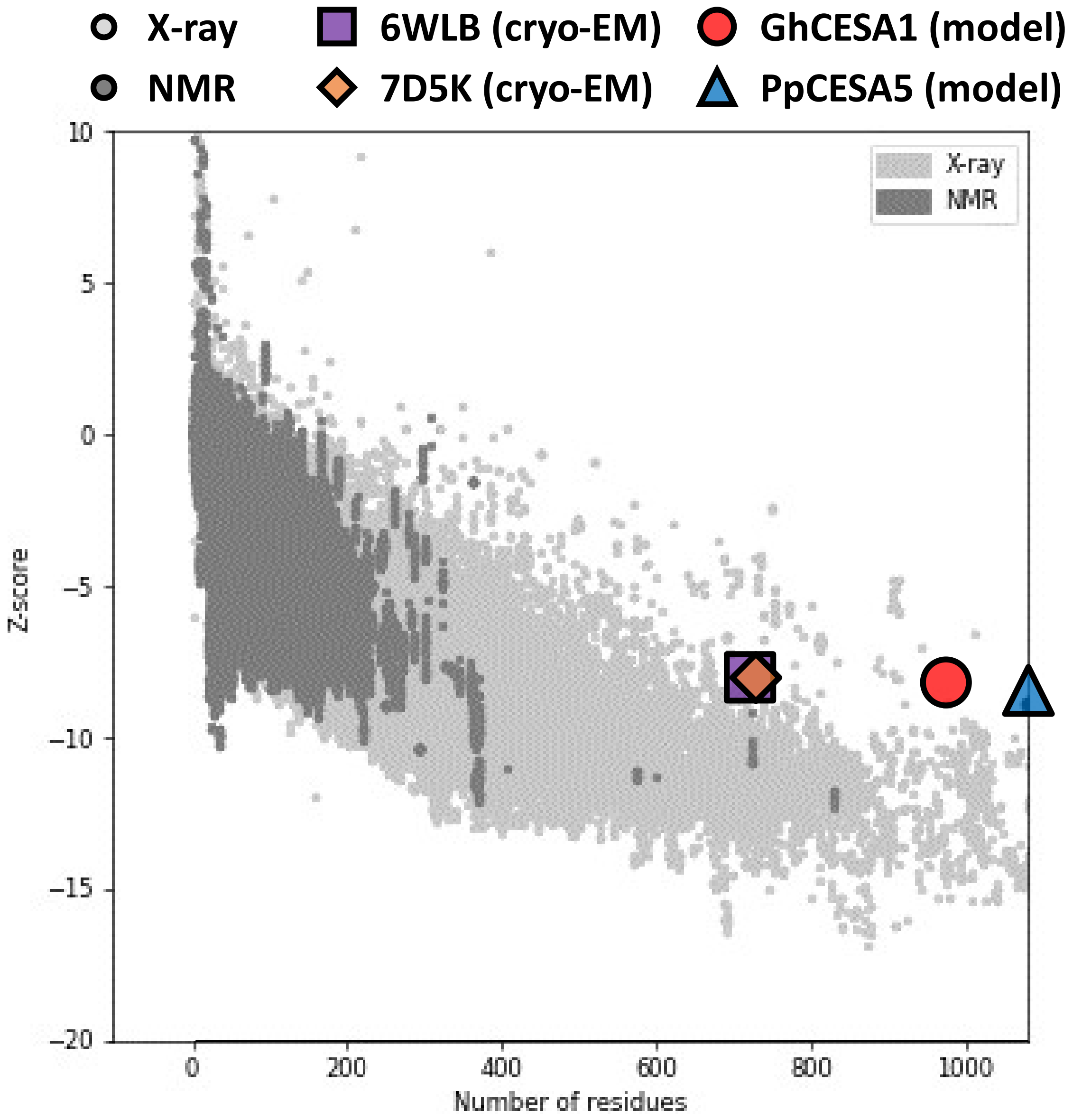

Fig. S2: ProSA-web z-score as a function of protein length (number of amino acid residues). ProSA-web z-scores are shown for two cryo-EM trimeric structures (6WLB PttCESA8, purple square; 7D5K GhCESA7, orange diamond), a hybrid GhCESA1 monomeric model after MD and post-MD energy minimization (GhCESA1, red circle), and a PpCESA5 trimeric model after MD and post-MD energy minimization (PpCESA5, blue triangle). For the data points representing trimers, the averaged ProSA-web z-score of the three monomers is plotted. These data points are superimposed onto a plot image, obtained from the ProSA-web server, containing data points from a set of X-ray and NMR structures from the Protein Data Bank.

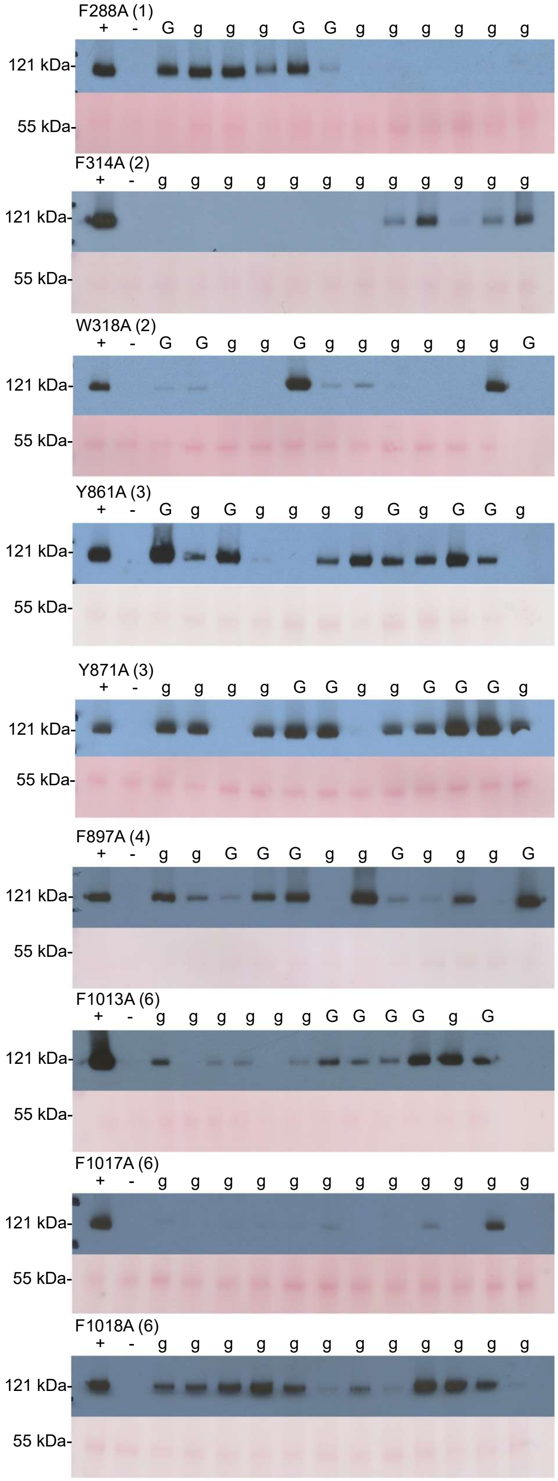

Fig. S3: Western blot analysis of protein expression for *P. patens* lines derived from transformation of *ppcesa5/6/7*KO protoplasts with vectors driving expression of PpCESA5 carrying the indicated mutations (Fig. 3). Western blots probed with anti-HA are shown above the same blot stained with Ponceau S as a loading control. Protein loading per lane was 7.0 µg. The 'G' or 'g' labels indicate lines that did or did not produce gametophores, respectively. Positive (+) and negative (-) control lines are included.

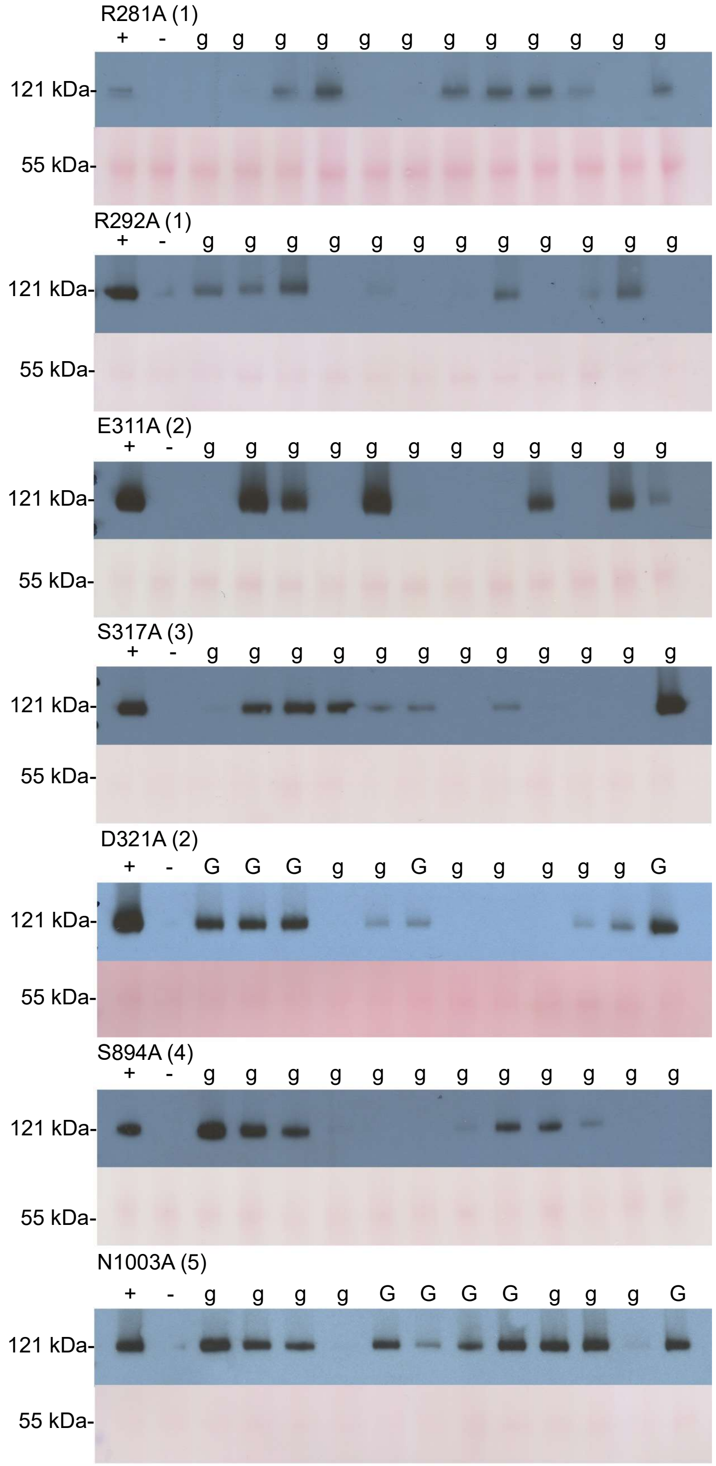

Fig. S4: Western blot analysis of protein expression for *P. patens* lines derived from transformation of *ppcesa5/6/7*KO protoplasts with vectors driving expression of PpCESA5 carrying the indicated mutations (Fig. 4). Western blots probed with anti-HA are shown above the same blot stained with Ponceau S as a loading control. Protein loading per lane was 7.0 µg. The 'G' or 'g' labels indicate lines that did or did not produce gametophores, respectively. Positive (+) and negative (-) control lines are included.

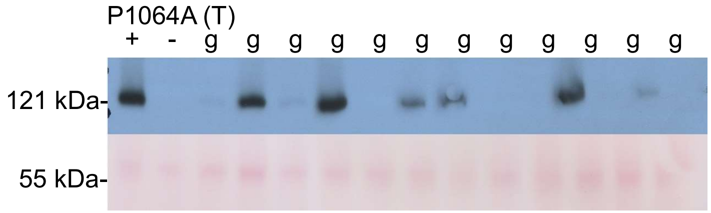

Fig. S5: Western blot analysis of protein expression for *P. patens* lines derived from transformation of *ppcesa5/6/7*KO protoplasts with vectors driving expression of PpCESA5 carrying the P1064A mutation (Fig. 5). Western blot probed with anti-HA is shown above the same blot stained with Ponceau S as a loading control. Protein loading per lane was 7.0 µg. Positive (+) and negative (-) control lines are included. None of these lines produced gametophores as indicated by lower case 'g'. Positive (+) and negative (-) control lines are included.

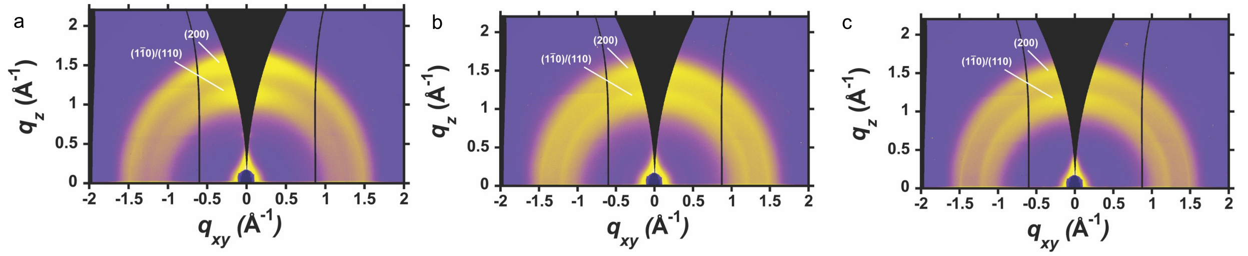

d

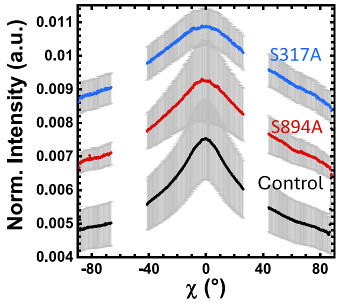

e

	n	Relative crystalline cellulose content (a.u.)	D-spacing of cellulose (200) (Å)	Coherence length of cellulose (200) (Å)
Control	3	1.17, 0.83, 1.00	4.02, 4.01, 4.05	31.0, 48.4, 29.7
S317A	3	1.88, 1.65, 0.66	4.06, 4.03, 4.06	30.4, 31.1, 29.5
S894A	3	2.06, 1.19, 1.63	4.06, 4.10, 4.06	31.3, 28.8, 28.3

Fig. S6: GIWAXS results from fully-expanded *P. patens* gametophore leaves (10 leaves per sample). a-c) Representative GIWAXS 2D data from stable lines selected from transformations of *cesa5*KO with wild-type control (a), S317A (b), or S894A (c) PpCESA5 expression vectors. d) Stitched χ-pole figures. Each trace is the average of two replicates for each of three independently selected lines (six traces per genotype), each normalized to the positive control for the respective beamtime. Gray areas are the standard error of the mean of replicate samples. e) Calculated values for relative crystalline cellulose content, d-spacing of cellulose, and coherence length of cellulose. Each value is for an independently selected line (average of two replicate measurements). All p values were > 0.05.

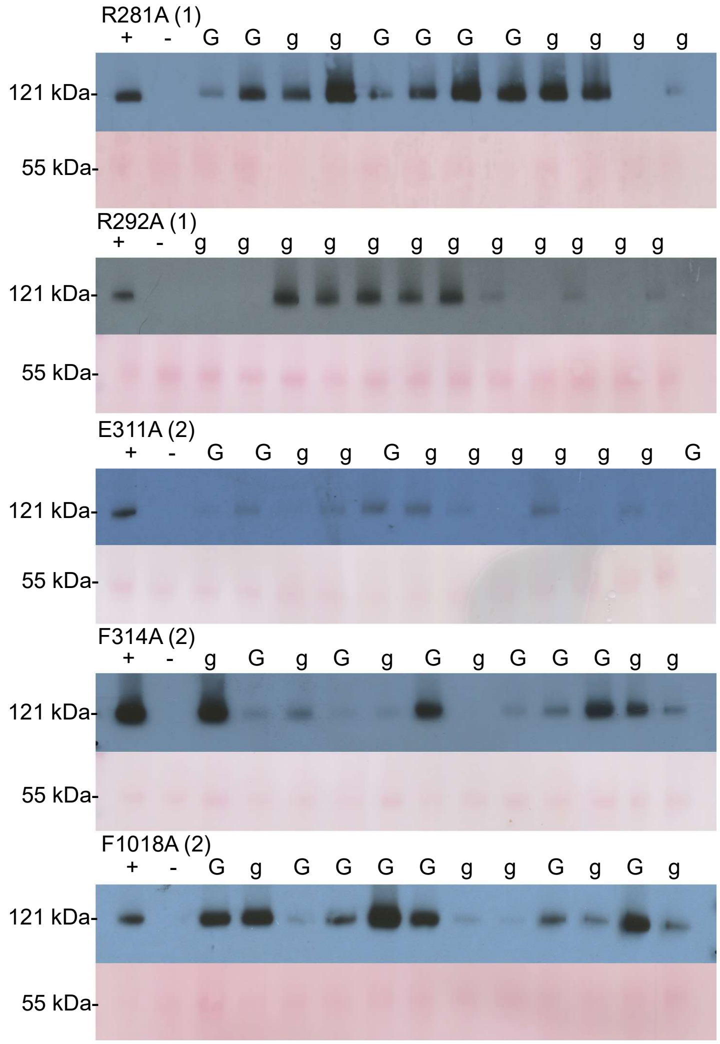

Fig. S7: Western blot analysis of protein expression for *P. patens* lines derived from transformation of *ppcesa5*KO protoplasts with vectors driving expression of PpCESA5 carrying the indicated mutations (Fig. 6). Western blots probed with anti-HA are shown above the same blot stained with Ponceau S as a loading control. Protein loading per lane was 7.0 µg. The 'G' or 'g' labels indicate lines that did or did not produce gametophores, respectively. Positive (+) and negative (-) control lines are included.

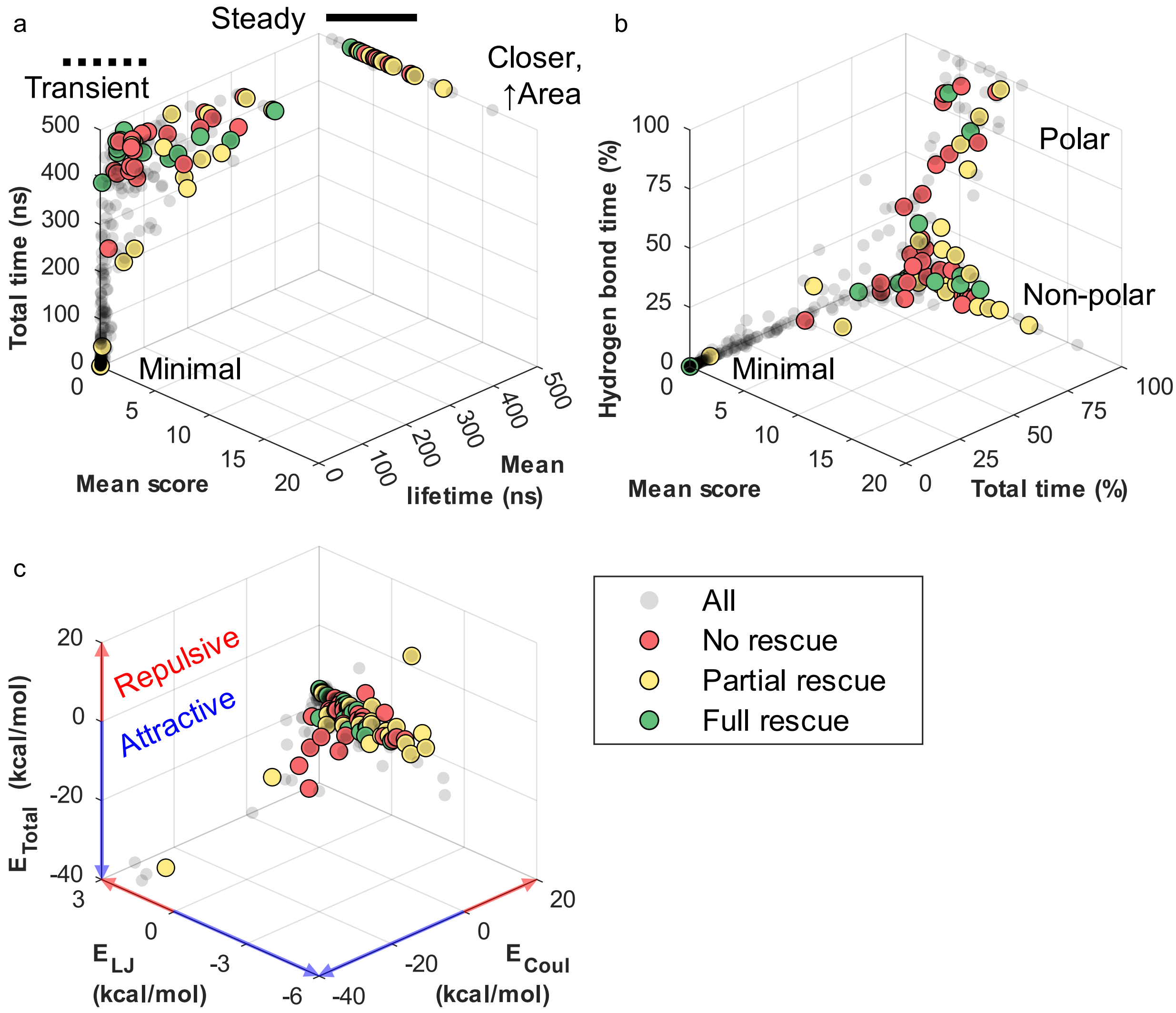

Fig. S8: Protein-glucan contact and energy metrics based on a 500-ns MD simulation of a PpCESA5 homotrimer. a) Mean score, mean lifetime, and total time. b) Mean score, total time, and hydrogen bond time, with hydrogen bond time expressed as a percentage relative to the total 500 ns of simulated time. c) Lennard-Jones, Coulombic, and total non-bonded interaction energies. Each data point represents one of 356 protein-glucan contacts, and 23 selected contacts are colored red (no rescue), yellow (partial rescue), or green (full rescue). Qualitative clusters or regions are indicated with annotations.

a

b

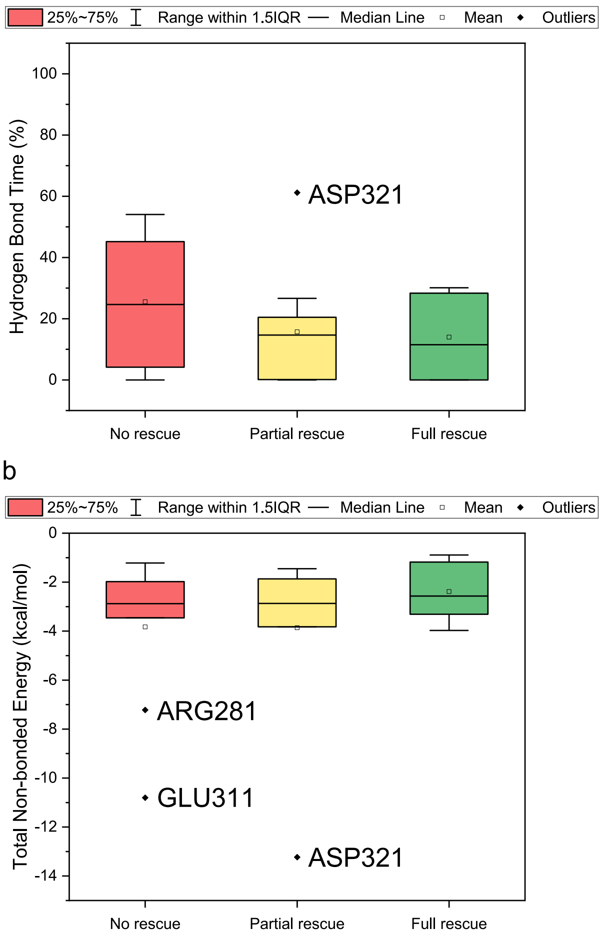

Fig. S9: Associations between contact metrics, energy metrics, and phenotype class. Box plots include the median, 25th and 75th percentiles, 1.5*IQR (interquartile range), mean, and statistical outliers. Phenotype classes include no rescue (red), partial rescue (yellow), and full rescue (green). a) Hydrogen bond time. b) Total non-bonded interaction energy.

```
RsBcsA    KMVPRFLLLSAASMLVMRYWFWRLFETL---PPPALDASFLF-ALLLFAVETFSISIFFLNGFLSADPTDRPFPR----P--LQ--   135
PttCESA8  KLTPYRAVIIMRLIILGLFFHYRITNPVDSA---------FGLWLTSVICEIWFAFSWVLDQFPKWKPVNRET--FIERLSARYER   243
GhCESA7   KINPYRMVIVARLLILAFFLRYRILNPVHDA---------IGLWLTSVICEIWFAFSWILDQFPKWFPIDRET--YLDRLSLRYER   323
PpCESA5   RINPYRMLIVIRLVVLAFFFRYRILNPVEGA---------YGMWLTSVICEIWFAISWILDQFPKWLPINRET--YLDRLSLRYEK   344
          *  *    *             *                     *     *        *  *

RsBcsA    ----PEELPTVDILVPSYN---EPADMLSVTLAAAKNMIYPARLRTVVLCDDGGTDQRCMSPDPELAQKAQERRRELQQLCR-EL-   212
PttCESA8  EGEPSQ-LAAVDFFVSTVDPLKEPPLITANTVLSILAVDYPVDKVSCYVSDDGA------------------AMLTFESLVETAE   309
GhCESA7   EGEPNM-LAPVDIFVSTVDPMKEPPLVTANTVLSILAMDYPVDKISCYISDDGAS------------------MLTFESLSETAE   389
PpCESA5   EGEPSQ-LEHVDIFVSTVDPMKEPPLVTANTILSILAVDYPVDKVSCYLSDDGAA------------------MLTFECISETSE   410

RsBcsA    --------------------------------------------------------------------------------------   212
PttCESA8  FARKWVPFCKKFSIEPRAPEFYFSQKIDYLKDKVQPSFVKERRAMKRDYEEYKVRVNALVPKAQKTPDEGWTMQDGTPWPGNNTRD   395
GhCESA7   FARKWVPFCKKFAIEPRAPEMYFTLKVDYLKDKVQPTFVKERRAMKREYEEFKVRINALVAKAQKVPPEGWIMQDGTPWPGNNTKD   475
PpCESA5   FARKWVPFCKKFSIEPRAPEMYFAQKIDYLKDKVQPTFVKERRAMKREYEEFKVRVNALVAKAQKVPEEGWTMQDGTPWPGNNSRD   496

RsBcsA    -----------------------GVVYSTRERN-----EHAKAGNMSAALERLK----GELVVVFDADHVP-SRDFLARTVGYFVE   265
PttCESA8  HPGMIQVFLGNTGARDIEGNELPRLVYVSREK-RPGYQHHKKAGAENALVRVSAVLTNAPYILNLDCDHYVNNSKAVREAMCILMD   480
GhCESA7   HPGMIQVFLGQSGGHDTEGNELPRLVYVSREK-RPGFLHHKKAGAMNALVRVSGVLTNAPFMLNLDCDHYINNSKAAREAMCFLMD   560
PpCESA5   HPGMIQVFLGHSGGHDTDGNELPRLVYVSREK-RPGFNHHKKAGAMNALVRVSAVLTNAPYFLNLDCDHYINNSKALREAMCFFMD   581

RsBcsA    ---DPDLFLVQTPHFFINPDPI----QRNLALGDRCP---PENEMFYGKIHRGLDRWGGAFFCGSAAVLRRRALDE----------   331
PttCESA8  PQVGRDVCYVQFPQRFDGI---DRSDR----------YANR-NIVFFDVNMKGLDGIQGPMYVGTGCVFNRQALYGYGPPSMPRLR   552
GhCESA7   PQIGRKVCYVQFPQRFDGI---DRHDR----------YANR-NTVFFDINMKGLDGIQGPVYVGTGCVFRRQALYGYEPPKGPKRP   632
PpCESA5   PSVGKKVCYVQFPQRFDGI---DRNDR----------YANH-NTVFFDINLKGLDGIQGPVYVGTGTVFNRKALYGYEPVLKEKES   653

RsBcsA    --------------------------------------------------------------------------------------   331
PttCESA8  KGKESS-SCFSCCCPTKKKPAQDPAEVYRDAK-----REDLNAAIFNLTEIDNYDDYERSMLISQLSFEKTFGLSSVFIESTLMEN   632
GhCESA7   KMVSCG-CCP--CFGRRKKDKKYPKN-----------GGNENGP--SLEAVED----DKELLMSQMNFEKKFGQSAIFVTSTLMDQ   698
PpCESA5   KGTGCGAACSTLCCGKRKKDKKKNKKSKFSRKKTAPTRSDSNIPIFSLEEIEEGDE-EKSSLVNTINYEKRFGQSPVFVASTLLEH   738

RsBcsA    -------------------------------AGGFAGETITEDAETALEIHSRGWKSLYIDR--AMIAGLQPETFASFIQQRGRW   383
PttCESA8  GGVPESANSSTLIKEAIHVIGCGFEEKTEWGKEIGWIYGSVTEDILSGFKMHCRGWRSIYCMPVRPAFKGSAPINLSDRLHQVLRW   718
GhCESA7   GGVPPSSSPAALLKEAIHVISCGYEDKTEWGSELGWIYGSITEDILTGFKMHCRGWRSIYCMPKLPAFKGSAPINLSDRLNQVLRW   784
PpCESA5   GGVHHSASPGSLLKEAIHVISCGYEDKTDWGKEIGWIYGSVTEDILTGFKMHCRGWRSIYCMPTRPAFKGSAPINLSDRLNQVLRW   824

RsBcsA    ATGMMQMLLL-KNPLFRR-G---LGIAQRLCYLNSMSFWFFPLVRMMFLVAPLIYLFF-GIEI-FVATFE--EVLAYMPGYLAVSF   460
PttCESA8  ALGSVEIFFSRHCPFWYGYGGGRLKWLQRLAYINTIVYPFTSLPLIAYCTIPAVCLLTGK---FIIPTLSNLA-SMLFLGLFISII   800
GhCESA7   ALGSVEIFFSRHCPAWYGLKGAKLRWLERFAYVNTTIYPFTSLPLLAYCTLPAICLLTDK---FIMPPISTFA-SLFFIALFLSIF   866
PpCESA5   ALGSVEISLSRHCPLWYGY-GGRLKCLERLAYINTTIYPLTSLPLVAYCVLPAVCLLTGN---FIIPTISNLD-SLYFISLFLSIF   905
                                           * *        *        *   *

RsBcsA    LVQNALFA---RQ---RWPLV-SEVYEVAQAPYL-ARAIVTTLLRPR----SARFAVTAKDETLSENYISP-------------IY   521
PttCESA8  VTAVLE-LRWSGVSIEDLW-RNE-QFWVIGGVSAHLFAVFQGFLKM-LAGIDTNFTVTAKAA---------DDTEFGELYMVKWT-   872
GhCESA7   ATGILE-LRWSGVSIEEWW-RNE-QFWVIGGISAHLFAVVQGLLKV-LAGIDTNFTVTSKTT---------DDEEFGELYTFKWT-   938
PpCESA5   VTGILE-MRWSGVGIDEWW-RNE-QFWVIGGVSAHLFALFQGLLKV-FAGVDTNFTVTSKQA---------DDEDFGELYMLKWT-   977

RsBcsA    --RP-LLFTFLLCLSGVLATLVRWVAF--PGDR-SVLLVVGGWAVLNVLLVGFALRAVAEKQQR----------------------   579
PttCESA8  TLLIPPTTLLIINIVGVVAGFSDALNKGYE--AWGPLFGKVFFAFWVILHLYPFLKGLMGRQNRTPTIVVLWSVLLASVFSLVWVK   956
GhCESA7   TLLIPPTTVLIINLVGVVAGISDAINNGY--QSWGPLFGKLFFSFWVIVHLYPFLKGLMGRQNRTPTIVVIWSVLLASIFSLLWVR  1022
PpCESA5   SLLIPPTTILILNLVGVVAGISDAINNGY--QSWGPLFGKLFFAFWVIVHLYPFLKGLMGRQNRTPTIVIVWSILLASIFSLLWVR  1061
                         ** *                 *                 *

RsBcsA    ----------------------                                                                  579
PttCESA8  INPFVNKVDNTLAGETCISIDC                                                                  978
GhCESA7   IDPFVLKTKG--PDTTQCGINC                                                                 1042
PpCESA5   INPFLSRSNG--PNLVECGLSC                                                                 1081
```

Fig. S10: Structure-based alignment of BsBcsA (4P00) and PttCESA8 (AF-Q6J8X0-F1) generated using the match→align tool in UCSF Chimera v. 1.16 with iteration until convergence across the alignment. GhCESA7 (Uniprot L7NUA2) and PpCESA5 (Phytozome Pp3c2_13330V1.1) sequences were added to the alignment using the consensus align tool (MUSCLE with 8 iterations) in Geneious Prime 2019.2.3. Small gaps in GhCESA7 and PpCESA5 were adjusted manually to bring these sequences into register with PttCESA8 throughout the alignment. The poorly aligned N-terminal regions of all four sequences were deleted for clarity. Residue numbers (right column) correspond to the full-length sequences. Color coding of protein features is as follows: transmembrane helices (TMH), gray; plant-conserved region, aqua; class-specific region, lavender; interfacial helix 3, gold; gating loop, green. Putative TMH glucan-interacting residues are shown in blue, and acceptor-coordinating residues are shown in magenta as identified in RsBcsA (Morgan et al. 2013, Fig. 3b; Morgan et al. 2014, Fig. 6d; Knott et al. 2016, Fig. 6; Zimmer 2019, Fig. 4), PttCESA8 (Purushotham et al. 2020, Fig. 3C, S5) and GhCESA7 (Zhang et al. 2021, Fig. S9). Putative TMH glucan-interacting residues that align across RsBcsA and plants are in bold text. PpCESA5 residues characterized in this study are color-coded by complementation assay results: no complementation, red; partial complementation, yellow; full complementation, green). Amino acid identity within the TMHs is indicated by *.

Table S1: Structural quality metrics for 30-aa N-terminal domain fragments of PpCESA5. Three structural quality metrics are shown, including the ERRAT quality factor, ProSA-web z-score, and the QMEAN4 z-score for three structural prediction web servers (I-TASSER, RaptorX-Contact, and Phyre2-Intensive), each of which produced up to 5 models. The cells for the metrics are colored from less favorable (red) to more favorable (green). For the three structural quality metrics, a rank from 1 to 11 is included in parentheses. A mean rank calculated across the three structural quality metrics is included at the bottom of the table. RaptorX-Contact Model 2 was chosen for subsequent use due to its highest mean rank of 1.70.

Metric	Web server	Model 1	Model 2	Model 3	Model 4	Model 5
ERRAT quality factor	I-TASSER	100.00 (1)	100.00 (1)	100.00 (1)	72.73 (10)	95.24 (8)
	RaptorX-Contact	100.00 (1)	100.00 (1)	100.00 (1)	100.00 (1)	78.95 (9)
	Phyre2-Intensive	15.79 (11)				
ProSA-web z-score	I-TASSER	-2.60 (4)	-2.32 (6)	-2.14 (7)	-3.04 (2)	-2.79 (3)
	RaptorX-Contact	-1.69 (9)	-3.10 (1)	-1.25 (11)	-2.59 (5)	-1.86 (8)
	Phyre2-Intensive	-1.29 (10)				
QMEAN4 z-score	I-TASSER	-1.17 (7)	-2.69 (9)	-1.28 (8)	-4.56 (10)	-1.15 (6)
	RaptorX-Contact	0.60 (2)	-0.24 (3)	-0.30 (4)	0.62 (1)	-0.37 (5)
	Phyre2-Intensive	-5.90 (11)				
Mean rank	I-TASSER	4.00	5.30	5.30	7.30	5.70
	RaptorX-Contact	4.00 [1]	**1.70** [2]	5.30	2.30 [3]	7.30
	Phyre2-Intensive	10.70				

[1] RaptorX-Contact: Model 1 (2nd best QMEAN z-score).
[2] RaptorX-Contact: Model 2 (3rd best QMEAN z-score but closest to zero (mean of reference set), lowest ProSA-web z-score, & best ProSA-web local quality profile).
[3] RaptorX-Contact: Model 4 (best QMEAN z-score, 2nd lowest ProSA-web z-score within the RaptorX group, & lowest mean ERRAT probability).

Table S2: Global structural quality metrics. Metrics are reported from the ProSA-web, MolProbity, and ERRAT web servers for two cryo-EM structures (PttCESA8, 6WLB; GhCESA7, 7D5K), a GhCESA1 model, and the final PpCESA5 homotrimeric model; data for the two models were obtained after MD simulations and up to 100K steps of post-MD energy minimization. Each output for the trimers represents a mean ± standard deviation from chains A, B, and C. The color scale is from red (lower quality) to green (higher quality). The goal values/ranges are based on the MolProbity and ERRAT web servers.

	**PttCESA8 trimer (3x 720 aa)**	**GhCESA7 trimer (3x 727 aa)**	**GhCESA1 monomer (974 aa)**	**PpCESA5 trimer (3x 1081 aa)**	
**Metric**	**cryo-EM: 6WLB (A, B, C)**	**cryo-EM: 7D5K (A, B, C)**	**Model: MD + 100K EM (A)**	**Model: MD + 100K EM (A, B, C)**	**Goal**
ProSA-web z-score	-7.99 ± 0.05	-7.99 ± 0.01	-8.17	-8.42 ± 0.45	--
MolProbity score	1.64 ± 0.05	1.81 ± 0.01	0.91	0.96 ± 0.03	--
> Percentile	90th to 93rd	84th to 85th	100th	100th	100th
Clashscore	6.38 ± 0.59	6.14 ± 0.09	0.00	0.00 ± 0.00	--
> Percentile	87th to 92nd	90th	100th	100th	100th
Poor rotamers	0.11 ± 0.09%	0.79 ± 0.00%	0.94%	0.92 ± 0.33%	< 0.3%
Favored rotamers	99.57 ± 0.24%	97.78 ± 0.00%	96.10%	95.32 ± 0.49%	> 98%
Phi-psi outliers	0.00 ± 0.00%	0.00 ± 0.00%	1.34%	0.77 ± 0.19%	< 0.05%
Phi-psi favored	95.80 ± 0.14%	92.23 ± 0.00%	93.83%	93.36 ± 1.04%	> 98%
Rama distribution z-score, |z|	1.38 ± 0.05	0.11 ± 0.01	1.33	1.71 ± 0.09	|z| < 2
C-beta deviations	0.00 ± 0.00%	0.00 ± 0.00%	0.33%	0.53 ± 0.06%	0%
Bad bonds	0.00 ± 0.00%	0.00 ± 0.00%	0.00%	0.00 ± 0.00%	0%
Bad angles	0.05 ± 0.01%	0.00 ± 0.00%	0.32%	0.54 ± 0.01%	< 0.1%
ERRAT quality factor	81.41 ± 0.67%	83.52 ± 0.24%	93.93%	89.89 ± 1.76%	100%

Table S3: CESA-glucan contacts. The columns include amino acid (AA) residue names and IDs (blue = cationic, red = anionic, green = polar, gray = non-polar aromatic, white = non-polar aliphatic), CESA domains, phenotype class, CESA count from the trimer used for averaging, contact metrics, and energy metrics.

AA residue	Domain	Class	Count	Mean score	Mean life-time (ns)	Total time (ns)	Total time (%)	H-bond time (%)	CH-π time (%)	E_{Coul} (kcal/mol)	E_{LJ} (kcal/mol)	E_{Total} (kcal/mol)
ARG-281	TMH1	No rescue	3	2.46	225.30	472.50	94.50	45.17		-6.37	-0.85	-7.22
ARG-292	TMH1	No rescue	3	3.13	46.83	462.00	92.40	24.63		-0.06	-1.32	-1.38
GLU-311	TMH2	No rescue	3	4.00	173.36	472.50	94.50	46.60		-9.39	-1.42	-10.80
PHE-314	TMH2	No rescue	3	4.99	416.58	499.83	99.97	54.03	0.00	-0.64	-1.96	-2.61
SER-317	TMH2	No rescue	3	2.07	28.14	488.00	97.60	23.20		-1.12	-0.86	-1.98
SER-894	TMH4	No rescue	3	3.74	245.31	497.17	99.43	31.50		-1.65	-1.28	-2.93
PHE-1017	TMH6	No rescue	3	5.90	388.78	499.67	99.93	0.47	8.73	-0.16	-3.30	-3.46
PHE-1018	TMH6	No rescue	3	3.60	35.54	482.83	96.57	4.17	0.17	-0.85	-2.03	-2.88
PRO-1064	C-term	No rescue	3	1.53	8.90	364.17	72.83	0.00		-0.13	-1.09	-1.22
PHE-288	TMH1	Partial rescue	3	3.01	218.25	493.50	98.70	0.00	0.17	0.03	-1.48	-1.45
TRP-318	TMH2	Partial rescue	3	4.26	388.78	499.67	99.93	0.27	0.00	0.20	-2.95	-2.74
ASP-321	TMH2	Partial rescue	3	7.19	416.58	499.83	99.97	61.17		-12.79	-0.44	-13.23
TYR-861	TMH3	Partial rescue	3	5.72	416.58	499.83	99.97	26.63	1.10	0.54	-2.41	-1.87
TYR-871	TMH3	Partial rescue	3	4.70	416.58	499.83	99.97	20.47	0.00	-2.18	-1.64	-3.83
PHE-897	TMH4	Partial rescue	3	8.78	212.39	495.33	99.07	0.00	54.97	-0.14	-3.68	-3.82
ASN-1003	TMH5	Partial rescue	3	2.64	23.52	320.67	64.13	17.97		-1.95	-1.15	-3.10
PHE-1013	TMH6	Partial rescue	3	4.30	181.27	422.50	84.50	0.13	0.70	-0.57	-2.30	-2.87
ARG-1068	C-term	Partial rescue	3	2.99	9.72	226.33	45.27	14.67		-0.48	-1.37	-1.85
LEU-285	TMH1	Full rescue	3	0.70	9.74	442.00	88.40	0.00		-0.23	-0.95	-1.18
LEU-900	TMH4	Full rescue	3	0.98	22.24	480.17	96.03	0.00		0.31	-1.20	-0.89
GLY-1014	TMH6	Full rescue	3	4.13	346.92	496.33	99.27	30.10		-2.17	-1.14	-3.31
TRP-1021	TMH6	Full rescue	3	6.04	157.99	498.67	99.73	28.33	4.07	-1.52	-2.45	-3.97
ILE-1062	TMH7	Full rescue	3	2.27	28.28	309.33	61.87	11.50		-1.37	-1.20	-2.57

Table S4: Primers used in this study.

Primer name	Primer sequence (5'→3')	Method	Fragment amplified
GITMH1_R281A_F<br>GITMH1_R281A_R	TATCGTAATCgcaCTCGTTGTGCTCGCCTTCTTC<br>AGCATGCGGTAGGGGTTG	Q5	NA
GITMH1_L285A_F<br>GITMH1_L285A_R	TCTCGTTGTGgcaGCCTTCTTCTTC<br>CGGATTACGATAAGCATG	Q5	NA
GITMH1_F288A_F<br>GITMH1_F288A_R	GCTCGCCTTCgcaTTCCGCTACC<br>ACAACGAGACGGATTACG	Q5	NA
GITMH1_R292A_F<br>GITMH1_R292A_R	CTTCCGCTACgcaATTCTCAACCCCG<br>AAGAAGGCGAGCACAACG	Q5	NA
GITMH2_E311A_F<br>GITMH2_E311A_R	GTCATTTGTGcaATTTGGTTCGCTATC<br>GGATGTAAGCCACATGCC	Q5	NA
GITMH2_F314A_F<br>GITMH2_F314A_R	TGAGATTTGGgcCGCTATCTCATG<br>CAAATGACGGATGTAAGC	Q5	NA
GITMH2_S317A_F<br>GITMH2_S317A_R	GTTCGCTATCgCATGGATTTTG<br>CAAATCTCACAAATGACG	Q5	NA
GITMH2_W318A_F<br>GITMH2_W318A_R	CGCTATCTCAgcGATTTTGGATCAG<br>AACCAAATCTCACAAATGAC	Q5	NA
GITMH2_D321A_F<br>GITMH2_D321A_R	TGGATTTTGGcTCAGTTCCCG<br>TGAGATAGCGAACCAAATC	Q5	NA
CESA5attB5<br><br>GITMH3_F861A SR1	GGGGACAACTTTGTATACAAAAGTTGCG<br>ATGGAGGCTAATGCAGGCCTTAT<br>GACGTCAACGGAGCGATTGTGGTGTTG	PCR fusion	Fragment 1 CESA5 F861A
GITMH3_F861A SF1<br><br>CESA5attB2	CAACACCACAATCGCTCCGTTGACGTC<br>GGGGACCACTTTGTACAAGAAAGCTGGG<br>TACTAACAGCTAAGCCCGCACTCGAC	PCR fusion	Fragment 2 CESA5 F861A
CESA5attB5<br>GITMH3_F871A SR1	See above<br>GGAAGCACGCAGGCCGCAACAAGAGG	PCR fusion	Fragment 1 CESA5 F871A
GITMH3_F871A SF1<br>CESA5attB2	CCTCTTGTTGCGGCCTGCGTGCTTCC<br>See above	PCR fusion	Fragment 2 CESA5 F871A
CESA5attB5<br>TMH4_S894A_SR	See above<br>GGAGATGAAGTACAATGCATCCAGGTTGCTAAT	PCR fusion	Fragment 1 CESA5 S894A
TMH4_S894A_SF<br>CESA5attB2	ATTAGCAACCTGGATGCATTGTACTTCATCTCC<br>See above	PCR fusion	Fragment 2 CESA5 S894A
CESA5attB5<br>TMH4_F897A_SR	See above<br>GAGAAAGAGGGAGATAGCGTACAAACTATCCAG	PCR fusion	Fragment 1 CESA5 F897A
TMH4_F897A_SF<br>CESA5attB2	CTGGATAGTTTGTACGCTATCTCCCTCTTTCTC<br>See above	PCR fusion	Fragment 2 CESA5 F897A
CESA5attB5<br>TMH4_L900A_SR	See above<br>GAAAATCGAGAGAAATGCGGAGATGAAGTACAA	PCR fusion	Fragment 1 CESA5 L900A
TMH4_L900A_SF<br>CESA5attB2	TTGTACTTCATCTCCGCATTTCTCTCGATTTTC<br>See above	PCR fusion	Fragment 2 CESA5 L900A
GITMH5_N1003A_F<br>GITMH5_N1003A_R	CGATGCAATAgcaAACGGCTACCAGTCCTGGG<br>GAGATGCCGGCCACGACG	Q5	NA
CESA5attB5<br>TMH6newF1013A_SR	See above<br>GAAGAACAGCTTCCCAGCCAACGGACCCCAGGA	PCR fusion	Fragment 1 CESA5 F1013A
TMH6newF1013A_SF<br>CESA5attB2	TCCTGGGGTCCGTTGGCTGGGAAGCTGTTCTTC<br>See above	PCR fusion	Fragment 2 CESA5 F1013A
CESA5attB5<br>TMH6newG1014A_SR	See above<br>GGCGAAGAACAGCTTTGCGAACAACGGACCCCA	PCR fusion	Fragment 1 CESA5 G1014A
TMH6newG1014A_SF<br>CESA5attB2	TGGGGTCCGTTGTTCGCAAAGCTGTTCTTCGCC<br>See above	PCR fusion	Fragment 2 CESA5 G1014A
CESA5attB5<br>TMH6newF1017A_SR	See above<br>CACCCAGAAGGCGAAAGCCAGCTTCCCGAACAA	PCR fusion	Fragment 1 CESA5 F1017A
TMH6newF1017A_SF<br>CESA5attB2	TTGTTCGGGAAGCTGGCTTTCGCCTTCTGGGTG<br>See above	PCR fusion	Fragment 2 CESA5 F1017A
CESA5attB5<br>TMH6newF1018A_SR	See above<br>GATCACCCAGAAGGCTGCGAACAGCTTCCCGAA	PCR fusion	Fragment 1 CESA5 1018A
TMH6newF1018A_SF	TTCGGGAAGCTGTTCGCAGCCTTCTGGGTGATC	PCR	Fragment 2

CESA5attB2	See above	fusion	CESA5 1018A
CESA5attB5<br>TMH6newW1021A_SR	See above<br>GCAAGTGCACGATCACTGCGAAGGCGAAGAACA<br>G	PCR<br>fusion	Fragment 1<br>CESA5 W1020A
TMH6newW1021A_SF<br>CESA5attB2	CTGTTCTTCGCCTTCGCAGTGATCGTGCACTTG<br>See above	PCR<br>fusion	Fragment 2<br>CESA5 W1020A
GI_TMH7_I1062A_F<br>GI_TMH7_I1062A_R	GTGGGTGCGGgcCAACCCGTTT<br>AGGAGAGAGAAGATGGAGG	Q5	NA
CESA5attB5<br>*GI_cterm_SR	See above<br>TGCAGACAAAAATGCGTTGATCCGCACC	PCR<br>fusion	Fragment 1<br>CESA5 P1064A
GI_cterm_SR<br>*CESA5attB2	GCATTTTTGTCTGCATCCAATGGACCCAAC<br>See above	PCR<br>fusion	Fragment 2<br>CESA5 P1064A
GI_cterm_R1068A_F<br>GI_cterm_R1068A_R	GTTTTTGTCTgcGTCCAATGGACCCAACC<br>GGGTTGATCCGCACCCAC	Q5	NA

*These primers were designed to introduce two mutations. However, a clone with only the P1064 mutation was recovered, eliminating the need to design primers for introducing only P1064.

Supplementary References:

Åqvist J, Luzhkov VB, Brandsdal BO (2002) Ligand binding affinities from MD simulations. Acc Chem Res 35:358-365. doi:10.1021/ar010014p

Benkert P, Biasini M, Schwede T (2011) Toward the estimation of the absolute quality of individual protein structure models. Bioinformatics 27:343-350. doi:10.1093/bioinformatics/btq662

Biasini M, Schmidt T, Bienert S, Mariani V, Studer G, Haas J, Johner N, Schenk AD, Philippsen A, Schwede T (2013) OpenStructure: an integrated software framework for computational structural biology. Acta Crystallogr D Biol Crystallogr 69:701-709. doi:10.1107/S0907444913007051

BIOVIA (2021) Discovery Studio Visualizer. Dassault Systèmes, San Diego

Case DA, Aktulga HM, Belfon K, Ben-Shalom IY, Brozell SR, Cerutti DS, Cheatham TE, III, Cisneros GA, Cruzeiro VWD, Darden TA, Duke RE, Giambasu G, Gilson MK, Gohlke H, Goetz AW, Harris R, Izadi S, Izmailov SA, Jin C, Kasavajhala K, Kaymak MC, King E, Kovalenko A, Kurtzman T, Lee TS, LeGrand S, Li P, Lin C, Liu J, Luchko T, Luo R, Machado M, Man V, Manathunga M, Merz KM, Miao Y, Mikhailovskii O, Monard G, Nguyen H, O'Hearn KA, Onufriev A, Pan F, Pantano S, Qi R, Rahnamoun A, Roe DR, Roitberg A, Sagui C, Schott-Verdugo S, Shen J, Simmerling CL, Skrynnikov NR, Smith J, Swails J, Walker RC, Wang J, Wei H, Wolf RM, Wu X, Xue Y, York DM, Zhao S, Kollman PA (2021) AMBER 2021. University of California, San Francisco

Case DA, Ben-Shalom IY, Brozell SR, Cerutti DS, Cheatham TE, III, Cruzeiro VWD, Darden TA, Duke RE, Ghoreishi D, Giambasu G, Giese T, Gilson MK, Gohlke H, Goetz AW, Greene D, Harris R, Homeyer N, Huang Y, Izadi S, Kovalenko A, Krasny R, Kurtzman T, Lee TS, LeGrand S, Li P, Lin C, Liu J, Luchko T, Luo R, Man V, Mermelstein DJ, Merz KM, Miao Y, Monard G, Nguyen C, Nguyen H, Onufriev A, Pan F, Qi R, Roe DR, Roitberg A, Sagui C, Schott-Verdugo S, Shen J, Simmerling CL, Smith J, Swails J, Walker RC, Wang J, Wei H, Wilson L, Wolf RM, Wu X, Xiao L, Xiong Y, York DM, Kollman PA (2019) AMBER 2019. Univeristy of California, San Francisco

Chen VB, Arendall WB, III, Headd JJ, Keedy DA, Immormino RM, Kapral GJ, Murray LW, Richardson JS, Richardson DC (2010) MolProbity: all-atom structure validation for macromolecular crystallography. Acta Crystallogr D Biol Crystallogr 66:12-21. doi:10.1107/S0907444909042073

Colovos C, Yeates TO (1993) Verification of protein structures: patterns of nonbonded atomic interactions. Protein Sci 2:1511-1519. doi:10.1002/pro.5560020916

Darden T, York D, Pedersen L (1993) Particle mesh Ewald: An N·log(N) method for Ewald sums in large systems. J Chem Phys 98:10089-10092

Grimsley NH, Grimsley JM, Hartmann E (1981) Fatty acid composition of mutants of the moss *Physcomitrella patens*. Phytochemistry 20:151901524

Hooft RW, Sander C, Vriend G (1997) Objectively judging the quality of a protein structure from a Ramachandran plot. Comput Appl Biosci 13:425-430. doi:10.1093/bioinformatics/13.4.425

Hudson KL, Bartlett GJ, Diehl RC, Agirre J, Gallagher T, Kiessling LL, Woolfson DN (2015) Carbohydrate-aromatic interactions in proteins. J Am Chem Soc 137:15152-15160. doi:10.1021/jacs.5b08424

Jorgensen WL, Chandrasekhar J, Madura JD, Impey RW, Klein ML (1983) Comparison of simple potential functions for simulating liquid water. J Chem Phys 79:926-935

Joung IS, Cheatham TE, III (2008) Determination of alkali and halide monovalent ion parameters for use in explicitly solvated biomolecular simulations. J Phys Chem B 112:9020-9041. doi:10.1021/jp8001614

Kelley LA, Mezulis S, Yates CM, Wass MN, Sternberg MJ (2015) The Phyre2 web portal for protein modeling, prediction and analysis. Nat Protoc 10:845-858. doi:10.1038/nprot.2015.053

Knott BC, Crowley MF, Himmel ME, Zimmer J, Beckham GT (2016) Simulations of cellulose translocation in the bacterial cellulose synthase suggest a regulatory mechanism for the dimeric structure of cellulose. Chem Sci 7:3108-3116. doi:10.1039/C5SC04558D

Kwansa AL, Singh A, Williams JT, Haigler CH, Roberts AW, Yingling YG (2024) Structural determination of a full-length plant cellulose synthase informed by experimental and *in silico* methods. Cellulose 31:1429-1447

Le Grand S, Götz AW, Walker RC (2013) SPFP: Speed without compromise—A mixed precision model for GPU accelerated molecular dynamics simulations. Comp Phys Comm 184:374-380

Lee J, Cheng X, Swails JM, Yeom MS, Eastman PK, Lemkul JA, Wei S, Buckner J, Jeong JC, Qi Y, Jo S, Pande VS, Case DA, Brooks CL, III, MacKerell AD, Jr., Klauda JB, Im W (2016) CHARMM-GUI input generator for NAMD, GROMACS, AMBER, OpenMM, and CHARMM/OpenMM simulations using the CHARMM36 additive force field. J Chem Theory Comput 12:405-413. doi:10.1021/acs.jctc.5b00935

Maier JA, Martinez C, Kasavajhala K, Wickstrom L, Hauser KE, Simmerling C (2015) ff14SB: Improving the accuracy of protein side chain and backbone parameters from ff99SB. J Chem Theory Comput 11:3696-3713. doi:10.1021/acs.jctc.5b00255

Martinez L, Andrade R, Birgin EG, Martinez JM (2009) PACKMOL: a package for building initial configurations for molecular dynamics simulations. J Comput Chem 30:2157-2164. doi:10.1002/jcc.21224

Morgan JL, McNamara JT, Zimmer J (2014) Mechanism of activation of bacterial cellulose synthase by cyclic di-GMP. Nat Struct Mol Biol 21:489-496. doi:10.1038/nsmb.2803

Morgan JLW, Strumillo J, Zimmer J (2013) Crystallographic snapshot of cellulose synthesis and membrane translocation. Nature 493:181-186. doi:10.1038/Nature11744

Nishiyama Y, Langan P, Chanzy H (2002) Crystal structure and hydrogen-bonding system in cellulose Ibeta from synchrotron X-ray and neutron fiber diffraction. J Am Chem Soc 124:9074-9082. doi:10.1021/ja0257319

Purushotham P, Ho R, Zimmer J (2020) Architecture of a catalytically active homotrimeric plant cellulose synthase complex. Science 369:1089-1094. doi:10.1126/science.abb2978

Resemann HC, Lewandowska M, Gömann J, Feussner I (2019) Membrane lipids, waxes and oxylipins in the moss model organism *Physcomitrella patens*. Plant Cell Physiol 60:1166-1175. doi:10.1093/pcp/pcz006

Ryckaert J-P, Ciccotti G, Berendsen HJC (1977) Numerical integration of the cartesian equations of motion of a system with constraints: molecular dynamics of n-alkanes. J Comp Phys 23:327-341

Salomon-Ferrer R, Götz AW, Poole D, Le Grand S, Walker RC (2013) Routine microsecond molecular dynamics simulations with AMBER on GPUs. 2. Explicit solvent particle mesh ewald. J Chem Theory Comput 9:3878-3888. doi:10.1021/ct400314y

Scheurer M, Rodenkirch P, Siggel M, Bernardi RC, Schulten K, Tajkhorshid E, Rudack T (2018) PyContact: Rapid, customizable, and visual analysis of noncovalent interactions in MD simulations. Biophys J 114:577-583. doi:10.1016/j.bpj.2017.12.003

Schott-Verdugo S, Gohlke H (2019) PACKMOL-Memgen: A simple-to-use, generalized workflow for membrane-protein-lipid-bilayer system building. J Chem Inf Model 59:2522-2528. doi:10.1021/acs.jcim.9b00269

Schrödinger (2012) Maestro. Schrödinger, LLC, New York

Singh A, Kwansa AL, Kim HS, Williams JT, Yang H, Li NK, Kubicki JD, Roberts AW, Haigler CH, Yingling YG (2020) *In silico* structure prediction of full-length cotton cellulose synthase protein (GhCESA1) and its hierarchical complexes. Cellulose 27:5597-5616

Sippl MJ (1993) Recognition of errors in three-dimensional structures of proteins. Proteins 17:355-362. doi:10.1002/prot.340170404

Sobieraj M, Setny P (2021) Entropy-based distance cutoff for protein internal contact networks. Proteins 89:1333-1339. doi:10.1002/prot.26154

Sobolev OV, Afonine PV, Moriarty NW, Hekkelman ML, Joosten RP, Perrakis A, Adams PD (2020) A global Ramachandran score identifies protein structures with unlikely stereochemistry. Structure 28:1249-1258 e1242. doi:10.1016/j.str.2020.08.005

Steinegger M, Meier M, Mirdita M, Vohringer H, Haunsberger SJ, Soding J (2019) HH-suite3 for fast remote homology detection and deep protein annotation. BMC Bioinformatics 20:473. doi:10.1186/s12859-019-3019-7

Studer G, Tauriello G, Bienert S, Biasini M, Johner N, Schwede T (2021) ProMod3-A versatile homology modelling toolbox. PLoS Comput Biol 17:e1008667. doi:10.1371/journal.pcbi.1008667

Viloria JS, Allega MF, Lambrughi M, Papaleo E (2017) An optimal distance cutoff for contact-based Protein Structure Networks using side-chain centers of mass. Sci Rep 7:2838. doi:10.1038/s41598-017-01498-6

Wang S, Sun S, Li Z, Zhang R, Xu J (2017) Accurate de novo prediction of protein contact map by ultra-deep learning model. PLoS Comput Biol 13:e1005324. doi:10.1371/journal.pcbi.1005324

Waterhouse A, Bertoni M, Bienert S, Studer G, Tauriello G, Gumienny R, Heer FT, de Beer TAP, Rempfer C, Bordoli L, Lepore R, Schwede T (2018) SWISS-MODEL: homology modelling of protein structures and complexes. Nucleic Acids Res 46:W296-W303. doi:10.1093/nar/gky427

Wiederstein M, Sippl MJ (2007) ProSA-web: interactive web service for the recognition of errors in three-dimensional structures of proteins. Nucleic Acids Res 35:W407-410. doi:10.1093/nar/gkm290

Williams CJ, Headd JJ, Moriarty NW, Prisant MG, Videau LL, Deis LN, Verma V, Keedy DA, Hintze BJ, Chen VB, Jain S, Lewis SM, Arendall WB, III, Snoeyink J, Adams PD, Lovell SC,

Richardson JS, Richardson DC (2018) MolProbity: More and better reference data for improved all-atom structure validation. Protein Sci 27:293-315. doi:10.1002/pro.3330
Zhang X, Xue Y, Guan Z, Zhou C, Nie Y, Men S, Wang Q, Shen C, Zhang D, Jin S, Tu L, Yin P, Zhang X (2021) Structural insights into homotrimeric assembly of cellulose synthase CesA7 from *Gossypium hirsutum*. Plant Biotechnol J 19:1579-1587. doi:10.1111/pbi.13571
Zhang Y (2008) I-TASSER server for protein 3D structure prediction. BMC Bioinformatics 9:40. doi:10.1186/1471-2105-9-40
Zimmer J (2019) Structural features underlying recognition and translocation of extracellular polysaccharides. Interface Focus 9:20180060. doi:10.1098/rsfs.2018.0060
